# Randomized clinical trial of ventilator liberation with pressure support ventilation versus therapist-implement patient-specific weaning in prolonged weaning patients via tracheostomy

**DOI:** 10.1186/s12890-026-04341-9

**Published:** 2026-05-08

**Authors:** Tamás Dolinay, Dale Jun, Swetha Gogineni, Lillian Hsu, Abigail Maller, B. Corbett Walsh, Jeffrey Gornbein

**Affiliations:** 1 Department of Medicine, University of California Los Angeles, 10833 Le Conte Ave, Los Angeles, CA 90095 USA; 2 Barlow Respiratory Hospital, 2000 Stadium Way, Los Angeles, CA 90026 USA

**Keywords:** Prolonged mechanical ventilation, Prolonged weaning, Tracheostomy, Ventilator liberation, Long-term acute care hospital

## Abstract

**Background:**

Liberation from prolonged mechanical ventilation is challenging and its outcomes are poor. Patients who failed at least three spontaneous breathing trials, often referred to as prolonged weaning patients, are usually weaned with protocolized programs in specialized weaning units, but there are no standardized strategies to facilitate their ventilator liberation. The objective of this study was to compare the ventilator liberation rate of two common ventilator weaning programs.

**Methods:**

Tracheostomized patients with ongoing invasive mechanical ventilation for at least 21 day who were admitted to Barlow Respiratory Hospital for ventilator weaning were studied. Patients who passed spontaneous breathing trial on admission were excluded. In a prospective parallel group, non-blinded clinical study, patients were randomized to receive either the Pressure Support Ventilation (PSV) weaning program or the Therapist-Implemented Patient-Specific (TIPS) weaning program. Randomization was performed using a computer algorithm of block design. The primary outcome was ventilator liberation success. The secondary outcomes were hospital length of stay, physical recovery, discharge disposition and mortality. Significant hospital events were also compared between the groups.

**Results:**

*N* = 25 patients were studied in PSV and *N* = 26 in the TIPS group. Outcomes were reported for all patients. The liberation success rate at 30 days was 37.5% (standard error, SE = 9.9%) in the PSV and 46.2% (SE = 9.8%) in the TIPS group (*p* = 0.58, odds ratio, OR 1.42, RD 8.7%, 95% confidence interval, CI=-18.6-35.9). The liberation rate at discharge was 44% (SE = 9.9%) in the PSV group and 53.8% (SE = 9.8%) in the TIPS group (*p* = 0.54, OR:1.48, RD 9.8%, CI=-17.2-37.2%). The inpatient mortality was: PSV = 24% (SE 8.5%) and TIPS = 11.5% (SE 6.3%), *p* = 0.291, OR 0.413, RD=-12.5%, CI=-33.2-8.3%. We did not find a significant difference between the two ventilator weaning programs in any of our outcomes, but our study describes a very sick patient population. Continued weaning beyond 30 days had improved liberation success.

**Conclusions:**

Both weaning paths are equally beneficial for prolonged mechanical ventilation patients who undergo prolonged weaning.

**Trial registration:**

The trial was registered retroactively at ClinicalTrials.gov, NCT06976554.

**Supplementary Information:**

The online version contains supplementary material available at 10.1186/s12890-026-04341-9.

## Introduction

Mechanical ventilation (MV) is a life-saving technology supporting approximately 300,000 hospitalized patients in the USA annually [1]. While MV was initially designed for short-term care, it is estimated that 6% of all MV patients will require prolonged mechanical ventilation (PMV) beyond 21 days, most via tracheostomy [2]. Tracheostomies provide a safe long-term artificial airway opening allowing prolonged weaning [3] but it remains unknown if the time to tracheostomy placement impact ventilator weaning success in the PMV population [4]. Data collected in Massachusetts suggests that the number of chronically ventilated patients continues to grow and may impact 7.6/100,000 people in the USA today [5]. PMV patients are usually managed in specialized weaning units (SWU) at long-term acute care hospitals (LTACH) [6]. LTACHs specialize in ventilator weaning of tracheostomized patients; however, the cost of hospitalization can reach over $10,000 a day [7, 8]. Despite specialized care, the one-year risk of death is approximately 55% in PMV patients and only about 54% of patients [6, 9] are liberated from the ventilator [10]. Current literature focuses on patients who have good tolerance for spontaneous breathing trials (SBT), representing only about 20% of those on PMV [9]. For patients who continue to fail SBT, referred to as prolonged weaning patients [3], pressure support (PS)-based ventilator weaning is recommended [3]. While weaning success in this population has critical clinical and societal implications, it remains unclear what weaning modality provides the best outcomes [9, 11].

In our prospective clinical study we compared two MV liberation strategies: A. pressure support ventilation (PSV) with daily rests [12] and B. the Therapist-Implemented Patient-Specific (TIPS) [11] weaning pathway. Pathway steps are shown in Table S1 (PSV) and in Table S2 (TIPS) in the Supplementary file.

## Methods

### Patient population

Patients admitted to Barlow Respiratory Hospital (BRH) between Aug 30, 2023 and April 1, 2025 with assist control MV via tracheotomy were considered for enrollment in our clinical study. BRH is a LTACH serving Southern California. The detailed inclusion and exclusion criteria are listed in the Supplementary file. In brief, we excluded patients with A. hemodynamic instability on admission, B. positive end expiratory pressure (PEEP) ventilation > 5 cmH2O, C. pass of SBT on admission with PS≤8cmH2O, D. without written consent for participation and E. previous failed weaning attempt at BRH. Patients were referred to the study based on the above listed criteria by the BRH attending pulmonologist. Written informed consent was obtained directly from all patients or their legally authorized representative. The study was approved by the University of California Los Angeles Internal Review Board (IRB, #22-001420). A copy of the approved IRB approved protocol, minor changes from the original protocol, consent form, IRB incident log and IRB approval letter is shown in the Supplementary file.

### Prospective clinical study

We conducted a prospective parallel group randomized, non-blinded clinical study in which eligible patients were randomized to receive PSV or TIPS weaning. The study was retroactively registered (ClinicalTrial.gov, NCT06976554, registration date April 28, 2025, first patient enrollment August 30, 2023, last patient enrollment March 20, 2025) because it was initially started as a quality improvement project at BRH. It is only upon internal review; after finishing enrollment but before data analysis, we were advised to register as a clinical trial. Our study examines a chronically hospitalized patient population in a specialized facility with a specific clinical expertise in ventilator weaning. We believe this study could not have been started outside of BRH and clinical trial preregistration would not have affected the results. However, it is our plan to preregister any future clinical study that stems from this investigation. The study does not have a sponsor and it compares two standard of care weaning protocols without an experimental arm. There was no patient involvement in the design or conduct of the study. However, the study was conducted to the highest scientific standards to limit bias. The study design is shown in Figure S1 in the Supplementary file. Patients who disconnected from MV for 7 consecutive days were considered liberated and completed the study. Patients who did not complete the ventilator weaning by day 30 failed the protocol and also completed the study. If a patient failed a weaning step for more than 3 consecutive days, the protocol was restarted from step one as long as the attending pulmonologist felt that it was safe to restart. Weaning could continue beyond the study period. Patients were followed for their entire BRH stay. There was no deviation from the study protocol. The study adheres to the CONSORT guidelines and the CONSORT 2025 checklist is included as a Supplementary file with this manuscript.

### Outcomes

Our primary outcome was ventilator liberation success at 30 days. Our secondary outcomes were: weaning success at discharge, change in Perme ICU Mobility score [13] from admission to discharge, length of hospital stay (LOS), discharge disposition, in-patient mortality and 90-day mortality. Significant hospital events were: weaning restarts, ICU admissions at BRH, transfer for higher level of care to short acute care hospital (STACH), tracheostomy decannulation, days to speaking valve use and to modified barium swallow test (MBST).

### Data collection

Demographics and comorbidities were collected from BRH medical records. The 90-day mortality after discharge was assessed by a phone call to the patient’s family. We were able to collect data from all patients during inpatient stay. There were 4 patients (*N* = 2 in PSV group and *N* = 2 in TIPS group) lost in follow up after discharge. We created a deidentified database which is available at osf.io. Data sharing statement is in the Supplementary file.

### Functional assessment and laboratory parameters

We measured the Perme ICU mobility score at admission and discharge to assess functional recovery [13]. Score elements are shown in the Supporting information S1 appendix. We also calculated the admission arterial partial oxygen pressure (PaO2) to the fraction of inspired oxygen (FiO2) ratio (PaO2/FiO2 ratio), the Blood Urea Nitrogen (BUN) to serum creatinine (Cr) ratio (BUN/Cr ratio) and blood platelet (Plt) count. Values were compared between groups.

### Statistical methods

Bivariate analysis: We used Fisher’s exact test to calculate the *p*-value between the two patient groups for categorical variables (liberation success, decannulation, ICU admission, death, discharge disposition, co-morbidities, gender, ethnicity, race and early tracheostomy). The p-values for comparing continuous variables between the two groups were computed either using the non-parametric Wilcoxon rank sum test if the data did not follow the normal distribution (time to tracheostomy, LOS, time to speaking valve use, number of weaning restarts, time to MBST, Perme score, age, PaO2/FiO2 ratio, BUN/Cr ratio and Plt count) or with t-tests if the data followed a normal distribution (age). If a continuous variable did not follow the normal distribution, the values are summarized with medians and the first and third quartile (interquartile range, IQR) instead of means and standard deviations (SD). Statistical significance was set at *p* < 0.05. For categorical variables we show the odds ratio (OR) and risk difference (RD) between PSV and TIPS weaning paths with 95 percentile confidence intervals for true RD.

## Results

### Patient selection

**Fig. 1:**
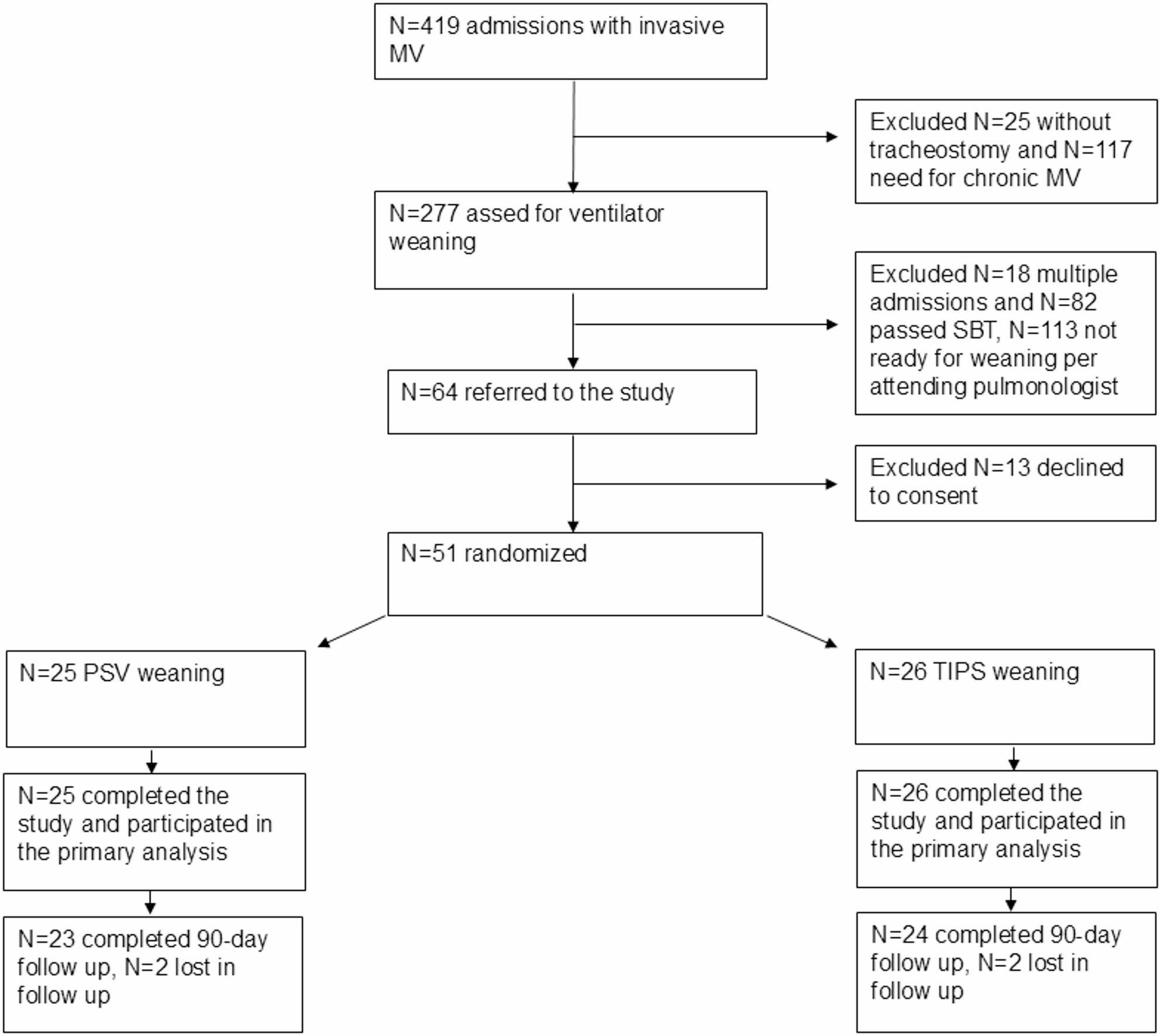
Study flow diagram. There were 419 admissions with invasive mechanical ventilation (MV) at Barlow Respiratory Hospital (BRH) between August 30th 2023 and April 1, 2025. Of all admissions 277 were assessed for ventilator weaning. *N*=64 was referred to the study by the BRH attending pulmonologist and 51 were enrolled in the study. *N*=25 patients were randomized to the Pressure support ventilation (PSV) weaning group and *N*=26 to the Therapist-Implemented Patient-Specific (TIPS) weaning group. All patients completed the study. *N*=2 patients were lost in 90-day follow up in both groups. Abbreviations: MV= invasive mechanical ventilation, SBT= spontaneous breathing trial, PSV= Pressure Support Ventilation, TIPS= Therapist-Implemented Patient-Specific

There were 419 admissions with invasive MV during the study period. Twenty-five did not have a tracheostomy and 117 were not considered for mechanical ventilator weaning because of long-term MV need. Of the 277 patients considered for ventilator weaning 259 were new admissions. Eighty-two patients passed SBT on admission (31.6%) and were started on unassisted breathing trial. One hundred seventy-seven patients failed SBT and of those 113 were not ready for ventilator weaning per the attending pulmonologist. Of the received 64 referrals, there were 51 patients recruited to the study, who were than randomized using a block design.The PSV path had 25 and the TIPS path had 26 patients. There were no withdrawals from the trial. The study flow diagram is shown in Fig. 1.

### Demographics, comorbidities and laboratory results

Baseline patient parameters are shown in Table 1. We did not find significant differences in the patient’s age (PSV mean=60yrs, SD = 16.9, TIPS mean = 76, SD = 11.24, *p* = 0.07), gender (female PSV = 28%, TIPS = 50%, *p* = 0.15), race (white PSV = 72%, TIPS = 85%, *p* = 0.35), and ethnicity (Hispanic PSV = 36%, TIPS = 19%, *p* = 0.22). We did not find clinically meaningful differences in 13 chronic and 13 acute comorbidities on admission between patient groups. We did not observe significant difference in the time to tracheostomy between the study group (median 25.3 days in the PSV group and 20.6 days in the TIPS group, *p* = 0.53). There were 7 chronic tracheostomy patients (5 in the PSV and 2 in the TIPS group). Of the remaining 44 patients, 9 tracheostomies were performed early (within 10 or less days from endotracheal intubations). There was no difference in ventilator liberation success in the early tracheostomy group when PSV and TIPS weaning were compared (15.8% vs. 24%, *p* = 0.71) We did not find a difference between admission PaO2/FiO2 ratio (median 293 in the PSV group and 309 in the TIPS group, *p* = 0.63), BUN/Cr ratio (median 29.4 in the PSV group and 29.5 in the TIPS group, *p* = 0.68) and Plt count (median 253 in the PSV group and 257 in the TIPS group, *p* = 0.55). Because we did not find significant differences in these values, we did not use them for statistical adjustment in our analysis.

**Table 1:** Patient demographics, chronic and acute comorbidities, predictors of weaning potential

	PSV = 25	TIPS = 26	*p*-value
demographics			
age (years, mean, SD)	66 (16.9)	74.6 (11.2)	0.07
gender (female, N, %)	7 (28)	13 (50)	0.15
race (white, N, %)	18 (72)	22 (84.6)	0.35
ethnicity (Hispanic, N, %)	9 (36)	5 (19.2)	0.22
chronic comorbidities			
diabetes (N, %)	8 (32)	14 (53.8)	0.16
hypertension (N, %)	13 (52)	21 (84)	0.03
chronic stroke (N, %)	9 (36)	9 (36)	0.99
coronary artery disease (N, %)	8 (32)	9 (36)	0.99
chronic kidney disease (N, %)	9 (36)	10 (38.5)	0.99
hemodialysis (N, %)	6 (24)	7 (26.9)	0.99
congestive heart failure (N, %)	9 (36)	13 (50)	0.4
chronic obstructive pulmonary disease (N, %)	3 (12)	3 (11.5)	0.99
pulmonary fibrosis (N, %)	2 (8)	2 (7.7)	0.99
body mass index > 35 (N, %)	6 (24)	6 (23)	0.99
neuromuscular disease (N, %)	9 (36)	4 (15.3)	0.19
chronic malignancy (N, %)	4 (16)	5 (19.2)	0.99
acute comorbidities			
cardiac arrest (N, %)	8 (32)	3 (11.5)	0.1
vasopressor need (N, %)	17 (68)	15 (57.7)	0.56
sepsis (N, %)	22 (88)	19 (73.1)	0.29
acute kidney disease (N, %)	13 (54.2)	11 (42.3)	0.57
acute stroke (N, %)	8 (32)	8 (30.8)	0.99
acute myocardial infarction (N, %)	3 (12)	0 (0)	0.23
acute hypoxemic respiratory failure (N, %)	25 (100)	25 (96.2)	0.99
pneumonia (N, %)	22 (88)	22 (84.6)	0.99
acute respiratory distress syndrome (N, %)	6 (24)	9 (34.6)	0.54
acute venous thromboembolism (N, %)	5 (20)	7 (26.9)	0.74
acute traumatic brain injury (N, %)	2 (8)	2 (7.7)	0.99
acute malignancy (N, %)	2 (8)	2 (7.7)	0.99
acute gastrointestinal bleed (N, %)	4 (16)	4 (15.4)	0.99
time to tracheostomy (days, median, IQR)	25.3 (12–27)	20.6 (10–24)	0.53
early tracheostomy (N, liberated %)	3 (15.8)	6 (24)	0.71
laboratory parameters			
paO2/FiO2 ratio (median, IQR)	293 (192–376)	309 (226–383)	0.63
BUN/Cr ratio (median, IQR)	29.4 (11.1–42.5)	29.5 (17.7–40.8)	0.68
platelet count (x10^3^/µl, median, IQR)	253 (228–302)	257 (202–365)	0.55

*Abbreviations*: *PaO2/FiO2 ratio* ratio of the arterial partial oxygen pressure (PaO2 in mmHg) to the fraction of inspired oxygen (FiO2 in %), *BUN/Cr* ratio of the blood urea nitrogen (BUN, mg/dl) ratio to serum creatinine (Cr, mg/dl), *PSV* pressure support ventilation weaning path, *TIPS *therapist-implemented patient-specific weaning path

### Ventilator liberation

**Fig. 2:**
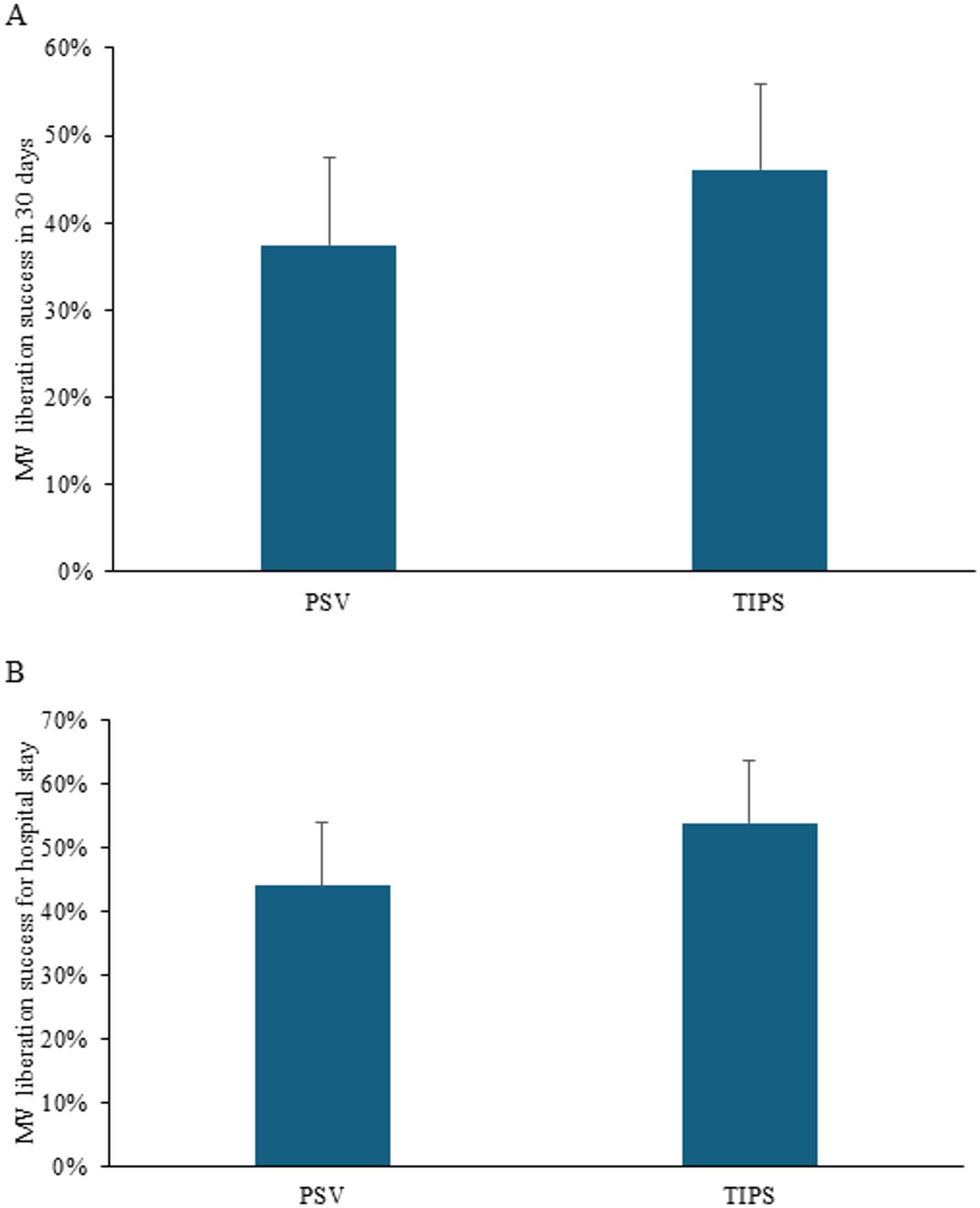
Ventilator liberation success from prolonged mechanical ventilation. **A**. Thirty-day liberation success. The liberation success was 37.5% (SE=9.9%) in the PSV and 46.2% (SE=9.8%) in the TIPS group. We did not find a difference in liberation success between PSV weaning and TIPS weaning protocols at 30 days (*p*=0.58, OR 1.42, RD 8.7%, CI=-18.6-35.9). **B**. Ventilator liberation success at the end of BRH hospitalization. The ventilator liberation success rate for the entire stay at BRH was 44% (SE=9.9%) in the PSV group and 53.8 (SE=9.8%) in the TIPS group. There was no significant difference between groups (*p*=0.54, OR:1.48, RD 9.8%, CI=-17.2-37.2%). Data is presented as % of success and standard of error (SE). Abbreviations: MV= invasive mechanical ventilation, SBT= spontaneous breathing trial, PSV= Pressure Support Ventilation, TIPS= Therapist-Implemented Patient-Specific

There was no difference in MV liberation between the PSV and the TIPS group. The ventilator liberation success during the 30-day study period (Fig. 2A) was 37.5% (SE = 9.9%) in the PSV group and 46.2% (SE = 9.8%) in the TIPS group (*p* = 0.58, OR 1.42, RD 8.7%, CI=-18.6-35.9). The ventilator liberation success rate for the entire stay at BRH (Fig. 2B) was 44% (SE = 9.9%) in the PSV group and 53.8% (SE = 9.8%) in the TIPS group (*p* = 0.54, OR:1.48, RD 9.8%, CI=-17.2-37.2%).

### Secondary outcomes

**Fig. 3:**
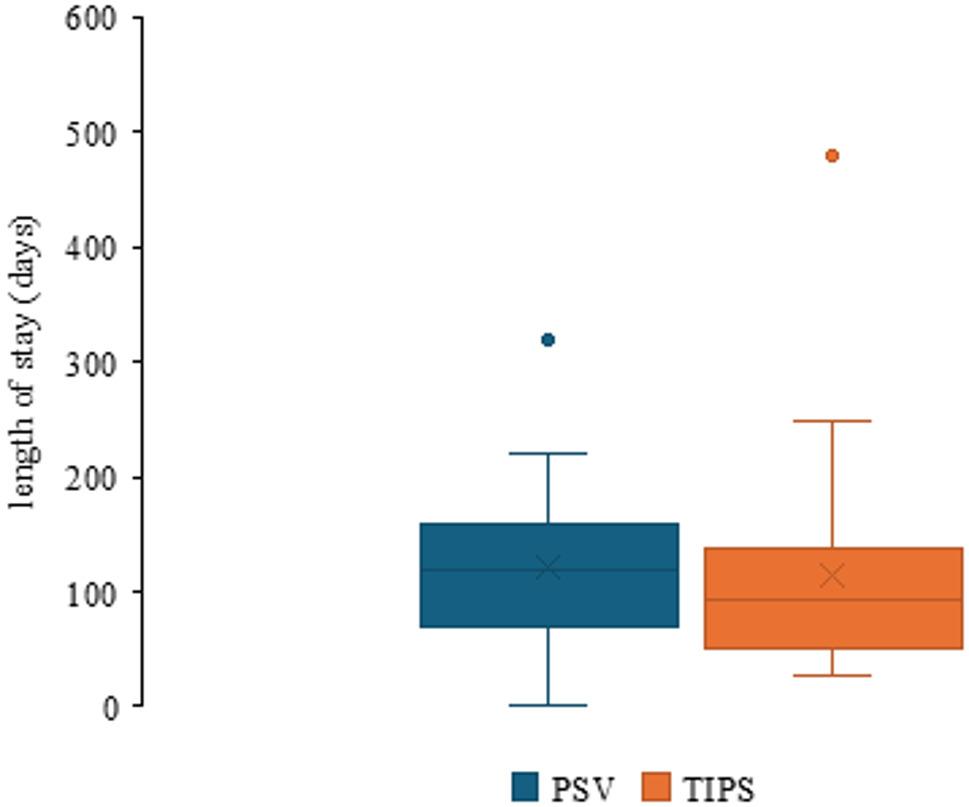
Prolonged hospital length of stay (LOS). No statistical difference noted between the groups. The LOS was a median of 121.5 days (IQR=85.5-157 days) for the PSV group and 93 days (IQR=52.2-134.5 days) for the TIPS group (*p*=0.19). Data is presented as median days with first to third interquartile range (IQR). Abbreviations: MV= invasive mechanical ventilation, SBT= spontaneous breathing trial, PSV= Pressure Support Ventilation, TIPS= Therapist-Implemented Patient-Specific

**Fig. 4:**
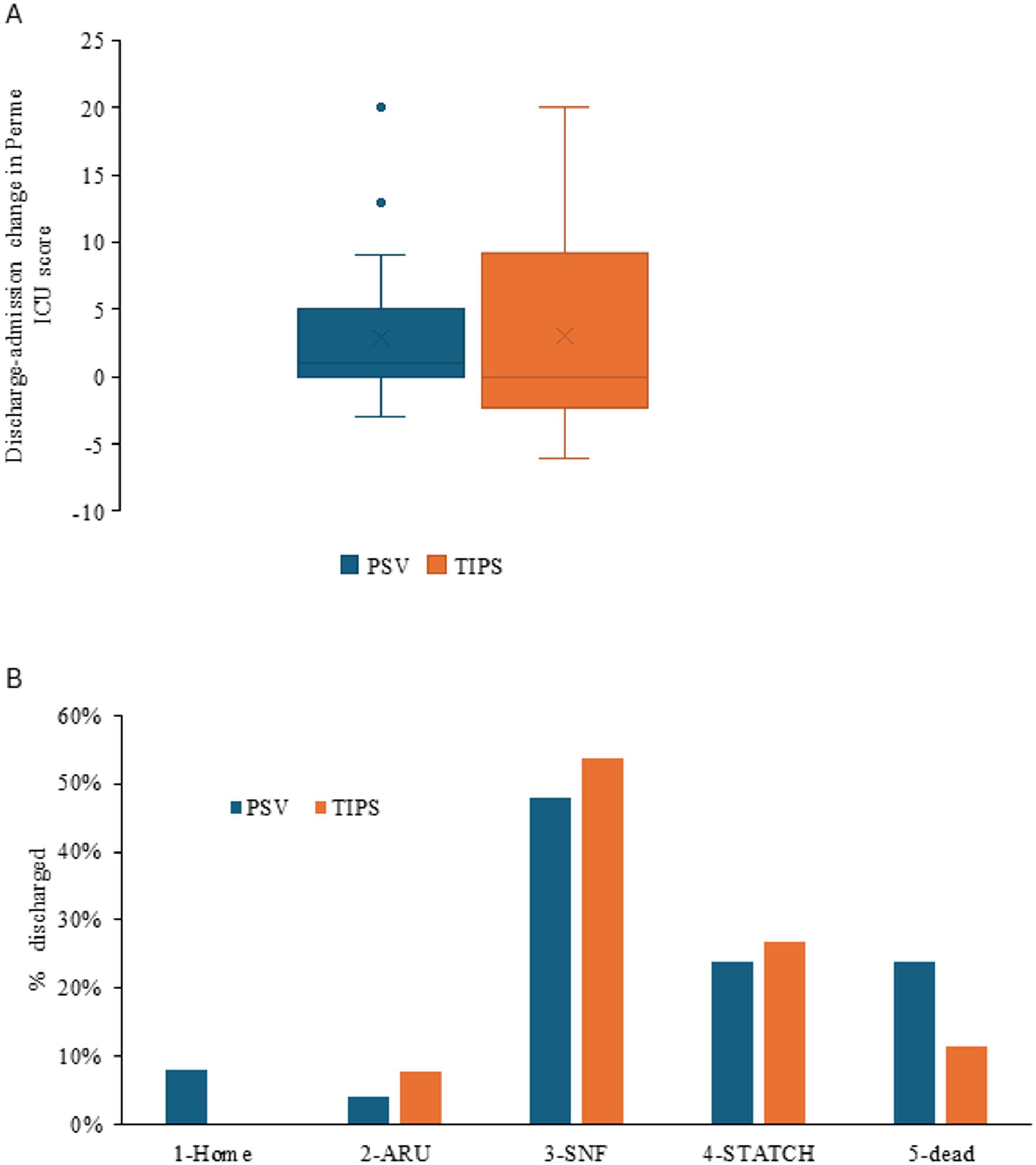
Poor functional recovery and disposition in prolonged mechanical ventilation with prolonged weaning. **A**. We calculated the difference between discharge and admission Perme ICU score for PSV and TIPS weaning protocol patients. Perme ICU score represents functional ability. There was a mean improvement of 3 in the score by discharge in both groups, but no significant differences between the two weaning paths (mean change=3, SD=5.3 for PSV and mean change=3, SD=7.5 for TIPS, *p*=0.36). **B**. Discharge to 5 potential locations. Discharge locations also signaled the long-term recovery potential. Home, acute rehabilitation unit (ARU), skilled nursing facility (SNF), short-term acute care hospital (STACH) transfer and death. The discharge disposition favored skill nursing facility (SNF) discharge for both weaning paths (48% in PSV group v 53.8% in the TIPS group, *p*=0.69). There was no significant difference in discharge locations between the two groups. Data presented as % of total discharges. Abbreviations: MV= invasive mechanical ventilation, SBT= spontaneous breathing trial, PSV= Pressure Support Ventilation, TIPS= Therapist-Implemented Patient-Specific

**Fig. 5:**
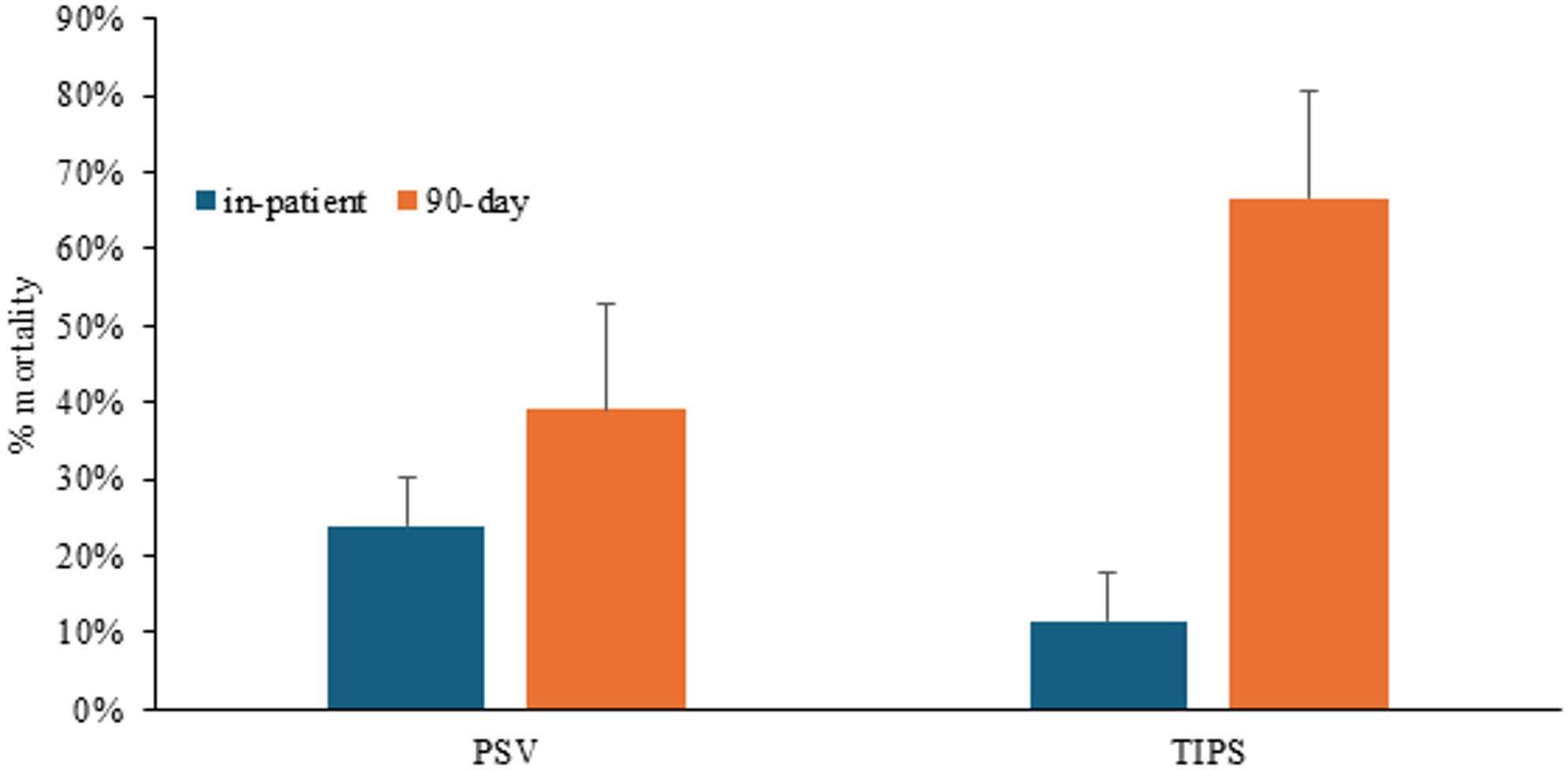
High mortality in prolonged mechanical ventilation with prolonged weaning. The in-patient mortality was 24% (SE 8.5%) in the PSV group and 11.5% (SE 6.3%) in the TIPS group (*p*=0.291, OR 0.413, RD=-12.5%, CI=-33.2-8.3%). The 90-day mortality was PSV=42.1% (SE=11.3%) and TIPS=66.7% (SE=9.6%) (*p*=0.162, OR 3.11, RD=27.5%, CI=-4.6-53.7%). There was a significant increase in mortality after the cessation of the TIPS weaning path (*p*=0.004) but not the PSV path (*p*=0.08). * Represents significant difference between in-patient and 90-day mortality for TIPS path. Abbreviations: MV= invasive mechanical ventilation, SBT= spontaneous breathing trial, PSV= Pressure Support Ventilation, TIPS= Therapist-Implemented Patient-Specific

There was no statistical difference in LOS between groups, but both the PSV and TIPS group had a prolonged LTACH hospitalization (Fig. 3). The LOS was a median of 121.5 days (IQR = 85.5–157 days) for the PSV group and 93 days (IQR = 52.2-134.5 days) for the TIPS group (*p* = 0.19). We did not find a significant difference in the change from admission to discharge Perme score between the two groups (Fig. 4A). The mean change was 3 for both groups (SD = 5.3 for PSV and 7.5 for TIPS, *p* = 0.36). We considered 5 possible discharge locations following BRH stay: (1) home, (2) acute rehabilitation unit (ARU), (3) skilled nursing facility (SNF), (4) STACH transfers and (5) death. There were only 2 discharges to home in the PSV group (8%) and no discharges to home in the TIPS group (0%). Patients with significant improvement in physical functioning were considered for ARU placement. There was 1 ARU discharge in the PSV group (4%) and 2 in the TIPS group (7.7%). The majority of patients in each group were admitted to SNF from BRH (48% in PSV group v 53.8% in the TIPS group, *p* = 0.69). No difference was found in discharge disposition between the two groups (Fig. 4B). We found a high mortality in both groups (Fig. 5). The inpatient mortality was: PSV = 24% (SE 8.5%) and TIPS = 11.5% (SE 6.3%), *p* = 0.291, OR 0.413, RD=-12.5%, CI=-33.2-8.3%. The 90-day mortality post discharge was very high in both groups (Fig. 5). The 90-day mortality was PSV = 42.1% (SE = 11.3%) and TIPS = 66.7% (SE = 9.6%), (*p* = 0.162, OR 3.11, RD = 27.5%, CI=-4.6-53.7%). There 90-day mortality was significantly higher in the TIPS path when compared to the in-patient mortality (11.5% vs. 66.7%, *p* = 0.004).

### Significant hospital events

We studied major events during BRH stay including major adverse events and progress with weaning (Table 2). The following major adverse events were considered: weaning restarts, ICU admission at BRH, transfers to STACH. BRH has a 6-bed ICU, but all major surgical and medical interventions require retransfer to a STACH. There were an average 1.6 ventilator weaning program restarts in the PSV group (SD = 1.66) and 1.3 restarts in the TIPS group (SD = 1.5), *p* = 0.66. There were 5 ICU admissions in the PSV group (20%) and 6 in the TIPS group (23%), *p* = 0.99. The STACH transfers were *N* = 6 in the PSV group (24%) and *N* = 7 in the TIPS group (26.9%) for higher level of care. To study the progress of ventilator weaning we recorded the days to speaking valve use, days to MBST, and the number of tracheostomy decannulations at BRH. The median time to speaking valve use was 2 days (IQR 0-11.25days) in the PSV group and 4 days (IQR 1–6 days) in the TIPS group (*p* = 0.74). Median time to MBST was 8 days in both groups (*p* = 0.94). Tracheostomy decannulation was completed in 2 patients (8%) in the PSV group and 3 patients (11.5%) in the TIPS group (*p* = 0.99).

**Table 2:** Significant hospital events during prolonged weaning

	PSV = 25	TIPS = 26	*p*-value
weaning restarts per patient (mean, SD)	1.6 (1.66)	1.3 (1.5)	0.66
ICU admission (N, %)	5 (20)	6 (23)	0.99
STACH transfer (N, %)	6 (24)	7 (26.9)	0.99
days to speaking valve use (median, IQR)	2 (0-11.25)	4 (1–6)	0.74
days to MBST (median, IQR)	8.0 (4.0–15.0)	8.0 (3.0–15.0)	0.94
tracheostomy decannulation (N, %)	2 (8)	3 (11.5)	0.99

*Abbreviations*: *ICU* intensive care unit, *MBST* modified barium swallow study, *PaO2/FiO2 ratio* ratio of the arterial partial oxygen pressure (PaO2 in mmHg) to the fraction of inspired oxygen (FiO2 in %), *BUN/Cr* ratio of the blood urea nitrogen (BUN, mg/dl) ratio to serum creatinine (Cr, mg/dl), *PSV* pressure support ventilation weaning path, *TIPS* therapist-implemented patient-specific weaning path

## Discussion

PMV, defined by more than 21 days of MV for at least 6 h a day, has poor clinical outcomes including severe disability, high infection risk and a mortality exceeding 50% within 1 year [14]. With the ever-rising care cost of hospitalization, PMV patients carry a significant burden on the health care system and their outcomes remain poor [15]. In the USA, and in many other countries, SWUs, commonly housed in a LTACH, take care of PMV patients with a lower cost [16]. Jubran et al. showed that about 54% all PMV patients can be liberated from MV in LTACHs and those who are liberated have an improved recovery potential [6]. In PMV patients, who tolerate SBT, unassisted breathing trials shorten the time to ventilator liberation, which is defined as 7 days without positive pressure ventilation [9, 17]. However in patients who failed SBT greater than 3 times, referred to as prolonged weaning patients [3], the mode of weaning is less well defined. Based on the seminal work of Brochard et al. [18] and Estaban et al. [12], protocolized PSV weaning is recommended is this population.

International guidelines recommend that assessment for ventilator liberation should start with a SBT as early as possible, but patient safety is critical [3]. Because the loss of PEEP during SBT can result in cardiopulmonary decompensation, a PEEP ≤ 8 cmH2O is felt to be a safe threshold to perform SBT. These recommendations were made for ventilator weaning with orally placed endotracheal tubes [3]. In patients with tracheostomies, which are usually larger and shorter than endotracheal tubes, a cutoff of PEEP ≤ 5 cmH2O is used [9, 19, 20]. However, it remains unknown if patients with a PEEP > 5 cmH2O can be safely enrolled in ventilator weaning programs. In our severely ill patient population, we did not attempt protocolized ventilator weaning with PEEP > 5 cmH2O, but we speculate that it would have compromised patient safety. In LTACHs, weaning protocols are part of a multidisciplinary rehabilitation program, which allows close monitoring and adjustment of weaning steps. The two most commonly used weaning paths are: A. PSV weaning with gradual decrease of ventilator pressure support (PS) interrupted with daily rest periods on assist control mode. When the patient tolerates low PS, repeated SBT are performed to assess for the feasibility of unassisted breathing trials. Unassisted breathing trials are performed with daily extensions of time without positive pressure ventilation and rest on assist control mode (Table S1 in the Supplementary file). The PSV weaning is usually 14 days. B. In TIPS weaning, the first 4 days require the patient to be closely watched on synchronized intermittent mandatory ventilation (SIMV) mode to assess for hemodynamic stability. This is followed by the gradual decrease of PS with a constant backup ventilator rate of 4 breaths/minute without rest periods. If a patient is able to tolerate SBT, they are monitored on SIMV mode between daily unassisted breathing trials (Table S2 in in the Supplementary file). The TIPS weaning is usually 21 days [11]. Despite their wide availability, to our knowledge, this is the first study comparing these two pathways.

Our primary outcome was ventilator liberation success. We found no significant difference between the two pathways. However, it is worth pointing out that there was an almost 10% risk difference in weaning success that favored TIPS. While acute hospital ICUs focus on rapid weaning, the primary objective in SWUs is preserving the patient’s hemodynamic stability during prolonged weaning. For patients who are unable to tolerate unassisted breathing trials, slower weaning may be beneficial. In these cases, TIPS may have the advantage over PSV because it is slower and warning signs of instability can be recognized and corrected earlier. Considering the time to discharge from BRH was over 3 months, regardless of liberation success or weaning pathway, taking the time to focus on patient stability was critical. Discharge timing was likely affected by the complexity of these patients, which limits their placement post discharge from the LTACH. Boles et al. in their expert document recommended that ventilator weaning should be continued for at least 3 months in PMV [3]. We agree with this statement based on our findings. Our data shows that there was an increase from 37.5% to 44% in liberation success in the PSV group and 44% to 53.8% in the TIPS group after the 30-day study period.

Low PaO2 [21], high BUN/Cr ratio [22] and low Plt count [23] have been shown to signal difficulty of ventilator weaning in PMV. In our study, admission PaO2/FiO2 ratio was within normal range, suggesting that the acute component of hypoxemic respiratory failure resolved by BRH admission. BUN/Cr ratio was elevated in both groups. This is consistent with the patient demographics that show close to 50% of our patients were survivors of acute renal failure and 25% of patients required chronic hemodialysis on admission. Low Plt count, which is common in acute critical illness and hematological malignancies, was rare in our patient population. We did not find differences in the admission values of these physiological indices between the two groups, indicating comparable disease severity.

The optimal timing of tracheotomy has been debated in the literature [24]. In some studies, early tracheostomy, performed within 10 days from endotracheal intubation, have shown reduction in the length of length of MV when compared to late tracheostomy, done after 10 days of MV [24–26]. However, the impact of tracheostomy timing on ventilator weaning in PMV patients remains unknown. Our study was not able to directly answer this question because ventilator weaning usually starts at the STACH and only those who failed initial weaning attempts were transferred to BRH. Our dataset noted no difference in ventilator liberation success between the two weaning programs in patients who had early tracheostomy.

Our results depict a very sick patient population. We have previously reported that BRH admits more complex patients for ventilator weaning, marked by a high case mix index, than the national average for LTACHs [27]. PMV patients are survivors of critical illness with various underlying pathology and disease acuity resulting in a heterogenous population. Recent research identified phenotypes of critically ill patients with ARDS and sepsis that show variable response to standard medical interventions and a subset of patients with a hyperinflammatory phenotype have worse clinical outcomes [28–30]. In our study, due to low enrollment, we could not perform subgroup analysis to identify factors that may lead to poor recovery potential and intolerance of ventilator weaning. In our future research, we would like to explore such factors by molecular and clinical phenotyping of PMV patients. While our patient population may represent a higher than usual disease severity, we believe our observations remain pertinent for general use in prolonged weaning. This is corroborated by our finding that 31.6% admitted for ventilator weaning were able to pass SBT on admission and our combined liberation success rate was 49%. There findings are similar to what was reported by Jubran et al. [6, 9].

The patients enrolled in our study showed a poor rehabilitation potential based on the Perme ICU mobility score, which was developed for critically ill patients [13]. Regardless of weaning path, 51% of patients required discharge to a SNF because of an ongoing medical need. Dubin et al. also found poor physical recovery in the LTACH for the chronically critically ill, but in their population 13% of patients were able to return home and 40% were discharged to an ARU [31]. Our study showed 8% returned home in the PSV and 0% in the TIPS group. There were 4% ARU discharges in the PSV group and 7.7% in the TIPS group. This may likely reflect that their patients were less sick than ours because patients with poor mental status were excluded [31]. We initially observed a 24% inpatient mortality in the PSV group and 11.5% in the TPS group during the 30 days of ventilator weaning. However, we noted a staggering 42% mortality in the PSV group and 66.7% mortality in the TIPS group 90 days post discharge. These findings are higher than reported by Kahn et al. but corroborating their statement that some LTACHs take care of sicker patients [32]. The familiarity of the BRH staff with ventilator weaning protocols played a critical role in identifying patients with early signs of deterioration, which in turn limited adverse events during the weaning process. We also speculate that the LTAHCs comprehensive care approach may keep the inpatient mortality lower.

We studied how in-hospital events shaped ventilator weaning. Major adverse events (ventilator weaning restarts, ICU admissions, higher level of care transfers and death) were common during ventilator weaning. Patients required at least 1 weaning restart (mean of 1.6 restarts in PSV versus 1.3 in TIPS). ICU admissions at BRH and transfers to higher level of care impacted approximately 25% of patients. However, ventilator restarts, transfer to STACH and in-patient mortality were not different between the groups. Recovery from tracheostomy is marked by ability to use a speaking valve, complete MBST and decannulation [31, 33]. There were no differences between PSV and TIPS that suggested either pathway was more successful to speed up tracheostomy recovery.

Many elements of the PSV and TIPS weaning program are similar. In fact, they are often used interchangeably or sequentially based on the experience of the institution and the pulmonologist. They both consist of PS reduction, followed by SBT and unassisted breathing trials. The major differences are: A. TIPS starts with 4 days of observation on SIMV mode with a reduction of backup rate, B. there are no daily rest period during PS reduction in TIPS, rather the patient is maintained on a backup rate of 4 breaths/minute, and C. the unassisted breathing trials are continued for 12 days in TIPS instead of 8 days in PSV weaning. We believe the reason the pathways performed similar in all indices is because they allow for constant supervision [34].

LTACHs have been criticized for prolonged stay, high-cost and high mortality [35–37]. Ventilator liberation success has also been low [6]. While acute hospitals can perform protocolized weaning as well as LTACHs, the care cost is significantly lower in the LTACH than in the acute care hospital [16]. SNFs can also execute successful weaning programs [37] but their resources are limited in addressing the complex care and complications arising from PMV. For many patients, LTACHs are the last comprehensive hospital environment where active interventions are performed. There is a growing movement to use LTACHs for palliative care consultations to help guide end of life decision making [38]. We believe LTACHs, will continue to be a critical piece of health care to support survivors of critical illness whose recovery cannot be determined during acute hospitalization.

Our study has several strengths. A. The major strength of our study is that it compares the PSV and TIPS weaning pathways in a randomized clinical study fashion limiting selection bias. B. BRH has ample experience with both weaning pathways, assuring weaning execution in a standardized manner. C. Lastly, we were able to follow patients during the entire hospitalization and post discharge, which provided us with a comprehensive evaluation of outcomes. However, our study has important limitations as well. A. It describes a weaning process in the post-acute care setting, which limits its applicability for acute care use. B. BRH admits higher acuity PMV patients, which makes it difficult to compare data amongst all weaning centers. C. Recruitment was difficult because survivors of critical illness with tracheotomy are generally unable to give consent and their families are weary to enroll in a randomized intervention. Approximately 40% (113 of 277 patients) of admissions were not initially considered for weaning, but may have started weaning later in the hospitalization. These patients were not captured in the study. Because of the low enrollment, we were unable to adjust for potential demographic and clinical confounders and we could not perform subgroup analyses. D. Finally, our study was performed at a single center specializing in ventilator weaning who was familiar with the studied weaning protocols. The weaning protocols were performed by the hospital staff as part of routine care, which prevented us from blinding them to the intervention. These inherent biases could limit the general applicability of our findings. To confirm our findings, we would to extend our research protocol to other LTACHs and weaning centers.

## Conclusion

PMV patients with prolonged weaning showed no significant difference between the PSV and TIPS pathway in MV liberation or in secondary outcomes. Repeated weaning trials from study completion to discharge resulted in additional ventilator liberation success of 6.5% in PSV and 7.6% in the TIPS pathway.

## Electronic Supplementary Material

Below is the link to the electronic supplementary material.

Supplementary Material 1.

## Data Availability

The research protocol and deidentified database is available immediately at: the Opens Science Frame Registries (OSF.IO). Study protocol is available at (10.17605/OSF.IO/VH4DF) and deidentified database with data library is available at 10.17605/OSF.IO/JMPRW.

## Abbreviations

ABG: Arterial blood gas
ARU: Acute rehabilitation unit
BRH: Barlow Respiratory Hospital
BUN: Blood urea nitrogen (mg/dl)
CI: 95% confidence interval
Cr: Serum creatinine (mg/dl)
FiO2: Fraction of inspired oxygen (%)
ICU: Intensive care unit
IQR: Interquartile range
LOS: Length of stay
LTACH: Long-term acute care hospital
MBST: Modified barium swallow test
MV: Mechanical ventilation
PaO2: Arterial partial oxygen pressure (mmHg)
PEEP: Positive end expiratory pressure (cmH2O)
Plt: Platelet count (blood, x10^3^/µl)
PMV: Prolonged mechanical ventilation
PS: Pressure support
PSV: Pressure Support Ventilation weaning
RD: Risk difference
SBT: Spontaneous breathing trial
SD: Standard deviation
SE: Standard of error
SIMV: Synchronized intermittent mandatory ventilation
SNF: Skilled nursing facility
STACH: Short-term acute care hospital
SWU: Specialized weaning unit
TIPS: Therapist-implemented Patient-specific weaning
