# Supplementary material for: Randomized clinical trial of ventilator liberation with pressure support ventilation versus therapist-implement patient-specific weaning in prolonged weaning patients via tracheostomy
Source: BMC Pulm Med. 2026 May 8;26:291. doi: 10.1186/s12890-026-04341-9 (PMC13321491; doi:10.1186/s12890-026-04341-9)

Randomized clinical trial of ventilator liberation with Pressure Support Ventilation versus Therapist-implement Patient-specific weaning in prolonged weaning patients via tracheostomy

Tamás Dolinay^1,2^, Dale June^1,2^, Swetha Gogineni^1,2^, Lillian Hsu^1,2^, Abigail Maller^1,2^, Brandon Corbett Walsh^1,2^, Jeffrey Gornbein^1^

^1^University of California Los Angeles Department of Medicine and ^2^Barlow Respiratory Hospital

**Supplementary file**

Content:

Study design-page 3

Figure S1-pages 4-5

Study protocol-pages 5-7

Inclusion and Exclusion criteria- pages 7-8

Changes in data collection from original internal review board protocol- pages 8-9

Patient randomization- page 9

Patient monitoring- pages 9-10

Patient safety and monitoring for adverse events- page 10

Perme ICU mobility score- pages 10-11

Data sharing statement- page 11

Ventilator weaning pathways- pages 11-14

Table S1- page 12

Table S2- page 14

UCLA IRB protocol -pages 15-37

UCLA IRB Consent Form- pages 37-45

IRB incident log- pages 46-48

IRB approval letter- pages 49-52

CONSORT 2025 checklist- pages 53-54

**Study design**

To study ventilator liberation success in prolonged mechanical ventilation (PMV) with tracheostomy for those who failed spontaneous breathing trial (SBT), the Pressure support ventilation (PSV) weaning path was compared to the Therapist-implemented Patient-specific (TIPS). Consented patients were randomized in two groups after admission to Barlow Respiratory Hospital (BRH). Both groups underwent a daily weaning protocol performed by a respiratory therapist. We followed patients for their entire length of stay. The program allows daily assessment for respiratory and hemodynamic function. The study design is shown in Figure S1.

Figure S1

liberated

**If failed 3x on consecutive days, reassess by pulmonologist for weaning. Can restart from Step 1 within 30 days. If cannot be liberated in 30 days or dead, study is completed**

can move up to 3 steps in one day

can move up to 3 steps in one day

4. Completed yesterday’s weaning step?

1. Hemodynamic and

respiratory status stable?

no

3. Completed daily weaning step starting Step1?

2. Attempt daily weaning

no

stable

study day

study protocol

BRH admission

D0

pass

consent

yes

D1-D30

randomize

**TIPS path**

fail

daily assessment

yes

liberated

no

yes

no

yes

no

yes

can repeat current step for up to 3 days

can move up to 3 steps in one day

**study completion**

exclusion (if the patient fails SBT on D2,

can be re-assessed for the study)

SBT

assess for hemodynamic/respiratory stability

unstable

**PSV pathway**

**TIPS pathway**

no

yes

yes

yes

no

no

yes

yes

no

no

yes

can repeat current step for up to 3 days, if passes continue path

liberated

**study completion**

Figure S1. Study design for the Randomized clinical trial of ventilator liberation with Pressure Support Ventilation versus Therapist-Implement Patient-Specific weaning in prolonged weaning patients via tracheostomy. Eligible patients were randomized to undergo the Pressure support ventilation (PSV) or Therapist-Implemented Patients-Specific (TIPS) weaning program. Ventilator liberation success was assessed at 30 days. Patients who were able to tolerate 7 consecutive days of unassisted weaning were considered liberated. If they were unable complete the weaning program by day 30 or died before 30 days, they were considered a weaning failure. If a patient was unable to complete the same step for 3 continuous days it indicated respiratory and/or hemodynamic instability and weaning was held. However, if a patient regained stability within the study period, the weaning was restarted from Step 1 per the attending pulmonologist’s direction. Patients were followed for their entire stay at Barlow Respiratory Hospital. Abbreviations: D0=Day0, D1-30=day 1 to 30. SBT= spontaneous breathing trial, PSV=pressure support ventilation weaning pathway, TIPS= Therapist-Implemented Patients-Specific ventilator weaning pathway

**Study protocol**

1. Patients were considered for enrollment in the study within 72 hours of admission to BRH.
2. Patients requiring positive pressure invasive mechanical ventilation (MV) underwent spontaneous breathing trial (SBT, pressure support (PS) ≤8cmH2O with 5 cmH2O of positive end expiratory pressure, PEEP for 1 hour) per hospital policy within 24 hours of admission. If they passed the SBT, they will be eligible for unassisted breathing trials (humidified, oxygenated air referred to as cool aerosol (CA) via tracheostomy collar without positive pressure mechanical ventilation). These patients are excluded from the study, except if they failed unassisted breathing trial within 4 hours and continued to require continuous positive pressure ventilation. Patients who failed unassisted breathing trial were considered for study enrollment the next day.
3. Patients required a tracheostomy to participate in the study.
4. Parameters to preclude study enrollment are listed in the Exclusion criteria section.
5. All patients were admitted to monitored beds at BRH. Vital signs (heart rate, oxygen saturation and lead 2 electrocardiogram, respiratory rate and ventilator compliance) were continuously monitored. Blood pressure is measured every 6 hours. Hemodynamic and respiratory stability was assessed daily by the nurse and the respiratory therapist. The list of monitored hemodynamic and respiratory parameters are shown in the Patient monitoring section.
6. On day 0 (D0), if a patient was stable and required continued MV, the study team approached the patient and/or the patient’s designated power of attorney (DPOA) for informed consent.
7. On D0, consented patients, were randomized to participate in the PSV or TIPS weaning pathways.
8. On day 1 (D1), weaning started. Hemodynamic and respiratory stability was assessed daily. In unstable patients the study was halted, but they will be followed throughout their hospitalization at BRH. Weaning was re-attempted within the study period if the patient was stabilized and the attending pulmonologist agreed to restart the weaning. Weaning was restarted from Step 1 in the same pathway.
9. If a patient failed to progress to the next step of ventilator weaning, the same step could be repeated on 3 consecutive days. If the subject was able to move to the next step, the weaning program continued.
10. If a patient could not restart the weaning within 30 days, the study was completed.
11. Both the PSV and TIPS pathway programs consist of MV and unassisted breathing parts. The length of the PSV pathway is14 steps and the length of TIPS pathway is 21 steps. These usually refer to days, but subjects were allowed to progress up to 3 steps a day. The details of both pathways are shown in the Ventilator weaning pathways section.
12. When a patient transitioned from MV to unassisted breathing, arterial blood gas (ABG), was collected after 2hrs of unassisted breathing to ensure adequate oxygenation and to avoid elevated carbon dioxide levels.
13. Following completion of the study all patients are assessed for tracheostomy decannulation by pulmonology, speech pathology and respiratory therapy.

**Inclusion and exclusion criteria**

The following inclusion and exclusion criteria were used for patient selection:

Subject Inclusion Criteria

1. Patients requiring positive pressure mechanical ventilation for at least 21 days prior to BRH admission and
2. Have a secure tracheostomy.

Subject Exclusion Criteria

1. Inability to obtain informed consent from patient or DPOA
2. Incarcerated patients
3. Patients with less than 3 months of life expectancy
4. Patients requiring vasopressor medication to stabilize blood pressure on admission
5. Systolic blood pressure less than 90mmHg on admission
6. Pulse less than 50 or greater than 130 beats per minute or change by more than 20 from baseline on admission
7. Respiratory rate greater than 35breaths/min
8. Oxygen saturation less than 90%
9. PEEP>5cmH2O
10. Lung tidal volume less than 250ml despite MV support
11. At least one previous admission to BRH with unsuccessful ventilator liberation attempt
12. Length of Stay (LOS) at BRH less than 24hours
13. Patients who passed SBT on D1 and eligible for unassisted breathing trial, except if they are unable to tolerate more than 4 hours of unassisted breathing time.

**Changes in data collection from original internal review board protocol**

We have made 3 minor changes in the data collection when compared to the originally submitted internal review board protocol. The changes were made before the study started and applied to all subjects.

1. We used the Perme ICU mobility score instead of the Functional Status Score for the Intensive Care Unit (FSS-ICU) score to access functional status, because the Perme ICU mobility score includes information mechanical ventilation need.

2. Ventilator liberation success was defined as 7 days of unassisted breathing instead of 3 days. Seven days of unassisted breathing is the generally accepted criteria for liberation success.

3. We did not limit the number of ventilator weaning restarts during the study period. In the original IRB submission, patients who failed 3 consecutive days of ventilator weaning were excluded from the study. This methodological change allowed us to count the number of adverse events leading to weaning halts and restart.

**Patient randomization**

Randomization was performed by a computer algorithm using block design in the patient database. The investigators did not have access to the randomization code. Once patient consent was obtained the investigators entered the patient information in the database and the computer system randomly assigned the patient to PSV or TIPS pathway protocols.

**Patient monitoring**

Patients were continuously monitored for stability throughout their entire hospital stay. The list of potential conditions listed here, lead to the hold of ventilator weaning.

1. Hemodynamic instability during ventilator weaning.
2. Patients requiring vasopressor medication to stabilize blood pressure
3. Systolic blood pressure less than 80mmHg
4. Pulse less than 50 or greater than 130 beats per minute or change by more than 20 from baseline on admission
5. Respiratory instability during ventilator weaning.
6. Respiratory rate greater than 35/min
7. Oxygen saturation less than 90%
8. PEEP>5cmH2O
9. Lung tidal volume less than 250ml despite MV support
10. Ventilator-patient desynchrony resulting in more than 2 discordant breaths per 10 seconds despite adjustment of ventilator pressure, flow or volume settings.
11. On ABG, pH<7.30, paO2<55mmHg, pCO2 >60 mmHg or no more than 10 mmHg change from admission baseline pCO2.
12. New onset fever, identified as core temperature >101F
13. Acute gastrointestinal bleed, identified as melena or bright blood per rectum.
14. Agitation, defined as pulling on tubes and line risking self-harm despite soft restraints.
15. Significant decline in cognition defined by unresponsiveness to command, if the patient was previously responsive.
16. Significant airway bleed resulting in suctioning difficulty of the airway

If a patients met one of the failed weaning criteria, the respiratory therapist returned the patient to full support ventilator support, alerted the attending pulmonologist and a member of the study team.

**Patient safety and monitoring for adverse events**

We followed the UCLA IRB guidelines for patient safety and monitoring for adverse events. The study had an independent study monitor, who reviewed all adverse events every 3 months. Major adverse were weaning restarts, ICU admissions, short-term acute care hospital (STACH) transfers and death.

**Perme ICU mobility score**

The Perme composite score was developed to assess physical functioning of ICU patients. It evaluates: 1. mentals status (0 to 3 points), 2. potential mobility barriers (0 to 4points), 3. functional strength (0 to 4points), 4. bed mobility (0 to 6points), 5. transfers (0 to 9 points), 6. gait (0-3 points) and 7. Endurance (0-3 points). The score scale is from 0 to 32 with the higher score representing higher functionality. We calculated the difference in the score between discharge and admission. The score instrumental details are available at [www.sralab.org/rehabilitation-measures/perme-icu-mobility-score](http://www.sralab.org/rehabilitation-measures/perme-icu-mobility-score).

**Data sharing statement**

Will individual data including data dictionary be available? Yes.

What particular data will be shared? Study protocol, deidentified database, data dictionary.

When will the data available? The data is available immediately at osf.io. No limit to data access.

With whom? Anyone who wishes to access the data?

For what type of analysis? Any type of analysis.

By what mechanism will the data be available? The data is immediately available at www.osf.io/jmprw/files/osfstorage

Ventilator weaning in patients with prolonged weaning via tracheostomy

**Ventilator weaning pathways**

PSV pathway (Table S1). In the PSV pathway patients completed a 14-step protocol. In this protocol gradual decrease of PS was used for 10-12 hours a day. PS was decreased from 20cmH2O to 10cmH2O with 5cmH2O PEEP applied (Step 1-6). Every weaning step was followed by rest on the same assist control ventilator setting the patient had on admission. On Step 7, patients underwent SBT (1 hour 5 cmH2O PS with 5cmH2O PEEP). If they passed, they progressed to unassisted breathing trials with CA via tracheostomy trach collar (Step 8-14). ABG was performed after 2 hours of unassisted breathing to assure respiratory stability. Respiratory stability was defined by pH > 7.30, paO2>55mmHg, pCO2<60 mmHg or no more than 10 mmHg change in pCO2 from admission baseline. Unassisted breathing time was extended by 4 hours daily in Step 8 to 14. Patients could progress up to 3 steps daily per the respiratory therapist and attending pulmonologist’s decision. The protocol completed when the patients was able to complete 72 hours of continuous CA time. Patients were monitored for an additional 4 days on the protocol to ensure ventilator liberation.

Table S1. PSV pathway protocol

| Step1. PS=20cmH2O PEEP=cm5H2O Backup rate=10* |
| --- |
| Step 2. PS=18 cmH2O PEEP=5 cmH2O Backup rate=10 |
| Step 3. PS=16 cmH2O PEEP=5 cmH2O Backup rate=10 |
| Step 4. PS=14 cmH2O PEEP=5 cmH2O Backup rate=10 |
| Step 5. PS=12 cmH2O PEEP=5 cmH2O Backup rate=10 |
| Step 6. PS=10 cmH2O PEEP=5 cmH2O Backup rate=10 |
| Step 7. SBT, if passed, CA for 4 hrs. (keep O2 sat>90%), Perform ABG after 2 hrs.# |
| Step 8. SBT, if passed, CA for 8 hrs. |
| Step 9. SBT, if passed, CA for 12 hrs. |
| Step 10. SBT, if passed, CA for 16 hrs. |
| Step 11. SBT, if passed, CA for 20 hrs. |
| Step 12. SBT, if passed, CA for 24 hrs. |
| Step 13. CA for 48hrs |
| Step 14. CA for 72 hrs.-protocol completed |
| Patient is monitored for 4 more days to ensure ventilator liberation |

* Weaning protocol is performed for 10-12 hours daily. Patients rested on admission assist control ventilator settings. # Rest on admission assist control ventilator settings between unassisted beathing trials. Abbreviations: PS=pressure support, PEEP=positive end expiratory pressure, SBT= spontaneous breathing trial, ABG arterial blood gas, CA= oxygenated cool aerosol via trach collar. The same abbreviations are used in the subsequent table.

TIPS pathway (Table S2). In the TIPS pathway, patients completed a 21-step protocol. On D1, admission mandatory ventilation mode was changed to synchronized intermittent ventilation (SIMV) and patients were challenged with SIMV settings: ventilator tidal volume (Vt) 6ml/kg, PS 20 cmH2O, PEEP 5cmH2O and a set respiratory rate (RR) 10 breaths/minute. During the first 4 steps the respiratory rate was gradually lowered from 10 breaths/minute to 4 breaths/minute (Step 1-4). Subsequently the PS was reduced from 20 to 10 cmH2O by each step (Step 5-9) in SIMV mode with a set RR=4 breath/minute. SIMV was continuous throughout Step 1 to 9. SBT was performed in Step 10 for 1 hour (5 cmH2O PS with 5cmH2O PEEP). If a patient passed SBT, it was followed with a 1hour unassisted breathing trial with CA using tracheostomy collar. ABG was performed in Step 11 following 2 hours CA time to ensure respiratory stability. Respiratory stability was defined as in the PSV pathway protocol. In steps 10-21, CA time was slowly increased by 1 to 4 hours and reached 24 hours by Step 19. Patients rested on SIMV Vt=6ml/h, PS=10 cmH2O, PEEP=5cmH2O, RR=4 breaths/minute (Step 9) setting between unassisted breathing trials. Patients could progress up to 3 steps daily as described in the PSV pathway. The protocol completed when a patient completed 72 hours continuous CA time. Patients were monitored for an additional 4 days on the protocol to ensure ventilator liberation.

Table S2. TIPS pathway protocol.

| Step 1. Transition from admission ventilator mode to SIMV Vt=6ml/kg, PS=20 cmH2O PEEP=5cmH2O, RR=10 |
| --- |
| Step 2. SIMV Vt=6ml/kg, PS=20 cmH2O PEEP=5cmH2O RR=8 |
| Step 3. SIMV Vt=6ml/kg, PS=20 cmH2O PEEP=5cm H2O RR=6 |
| Step 4. SIMV Vt=6ml/kg, PS=20 cmH2O PEEP=5cm H2O RR=4 |
| Step 5. SIMV Vt=6ml/kg, PS=18 cmH2O PEEP=5cm H2O RR=4 |
| Step 6. SIMV Vt=6ml/kg PS=16 cmH2O PEEP=5cm H2O RR=4 |
| Step 7. SIMV, Vt=6ml/kg, PS=14 cmH2O PEEP=5cm H2O RR=4 |
| Step 8. SIMV Vt=6ml/kg, PS=12 cmH2O PEEP=5cm H2O RR=4 |
| Step 9. SIMV Vt=6ml/kg, PS=10 cmH2O PEEP=5cm H2O RR=4 |
| Step 10. SBT, if passed, 1 hrs. CA rest on Step 9 settings |
| Step 11. SBT, if passed, 2 hrs. CA rest on Step 9 settings. ABG was performed |
| Step 12. SBT, if passed, 4 hrs. CA rest on Step 9 settings |
| Step 13. SBT, if passed, 6 hrs. CA rest on Step 9 settings |
| Step 14. SBT, if passed, 8 hrs. CA rest on Step 9 settings |
| Step 15. SBT, if passed, 10 hrs. CA rest on Step 9 settings |
| Step 16. SBT, if passed, 12 hrs. CA rest on Step 9 settings |
| Step 17. SBT, if passed, 16 hrs. CA rest on Step 9 settings |
| Step 18. SBT, if passed, 20 hrs. CA rest on Step 9 settings |
| Step 19. SBT, if passed, 24 hrs. CA |
| Step 20. 48 hrs. CA |
| Step 21. 72 hrs. CA- protocol completed |
| Patient is monitored for 4 more days to ensure ventilator liberation |

Abbreviations: SIMV=synchronized intermittent mandatory ventilation, RR= respiratory rate, Vt=ventilator tidal volume

**UCLA IRB**

Ventilator weaning in patients with prolonged mechanical ventilation

Primary Investigator: Tamas Dolinay MD., PhD

Co-investigators: Dale Jun MD, Abigail Maller MD, Augustine Chung MD, Lillian Hsu MD, Brandon Grimes MD, David Nelson MD

Statistician: Jeffrey Gornbein Dr.PH.

Funding UCLA Department of Medicine, Division of Pulmonary, Critical Care and Sleep medicine internal funding and Barlow Respiratory Hospital

1.0 PROTOCOL SUMMARY

Title:

Mechanical ventilator weaning in chronically ventilated patients

Objective:

To compare the success rate of two protocolized weaning programs in patients requiring prolonged mechanical ventilation (PMV). We will compare Pressure support ventilation (PSV) weaning protocol to the Therapist-Implemented Patient-Specific (TIPS) weaning protocol.

Patient population:

PMV patients admitted to Barlow respiratory Hospital (BRH), a long-term acute care hospital (LTACH) in Los Angeles, California, for ventilator weaning.

Protocol design:

Eligible patients will be randomly assigned to one of two ventilator weaning protocol paths: 1.PSV path. This protocol uses a daily schedule of gradual reduction of PSV combined with daily rests on mandatory ventilator mode. The PSV path is at least 14 days long. 2. TIPS path. This protocol uses a daily schedule of SIMV ventilation with gradual reduction of mandatory ventilator support followed by PSV weaning. The TIPS path is at least 21 days long.

Primary outcome:

Weaned from mechanical ventilation (MV) by 30 days from LTACH admission.

Time of completion:

Patients who can disconnect from MV for 3 consecutive days or have their tracheostomy decannulated will be considered weaned (“success”) and will have completed the protocol. Patient who cannot complete the ventilator weaning by 30 days will fail the protocol and complete the study.

2.0 OBJECTIVES AND SCIENTIFIC AIMS

Mechanical ventilation (MV) is a life-saving technology supporting approximately 300,000 hospitalized patients in the USA annually(1). While initially designed for short-term care, it is estimated that 6% of all MV patient will require PMV beyond 21 days(2). PMV is associated with increased mortality and the risk of death is approximately 55% in one year(3, 4). Data collected in Massachusetts suggest that the number of chronically ventilated patients continues to grow and may affect 7.6/100.000 people in the USA(5). Many PMV patients are cared for in long-term acute care hospitals (LTACH)(4), which specialize in ventilator weaning (weaning) of tracheostomized patients. Tracheostomies provide safe long-term artificial airway opening which allows slower weaning(6). The daily cost of care for PMV patient in the LTACH is greater than $10,000 (7, 8). Despite the specialized care, only about 54% of patients are liberated from the ventilator(9).While improving weaning success in the PMV population has critical clinical and societal implications, it is not known what is the best weaning modality for these patients(3, 10). Available literature has focused on patients with good tolerance for spontaneous breathing trials (SBT), but this likely represents only about 20% of all PMV patients(3). In PMV patients, protocolized ventilator weaning using pressure support-based ventilator weaning have been recommended(6) but it remains unclear what protocol is the most beneficial.

The overall objective of this study is to establish the weaning success rate in patients requiring PMV, who cannot tolerate SBT. We will compare two established weaning protocols: A. pressure support ventilation (PSV) weaning with daily rest periods(11) and B. the Therapist-implemented patient specific (TIPS) weaning, which combines synchronized intermittent mandatory ventilation (SIMV) and PSV weaning(10).

Our central hypotheses are 1. weaning success in PMV patients is related to preserved hemodynamical and respiratory stability during the weaning process rather than the method of ventilator weaning (the criteria for hemodynamical and respiratory stability is listed in Section 5.3) and 2. PSV weaning protocol path is non-inferior to TIPS weaning protocol path.

To test the central hypotheses and to attain the objective of this project we will pursue the following *Specific Aims*:

- Scientific aim 1. Compare the success rate of PSV and TIPS weaning paths in a randomized, non-blinded clinical trial. At the time of completion the study will be able to answer the question if the PSV path is non-inferior to the TIPS path in ventilator weaning.
- Scientific aim 2. Compare the number of tracheotomy decannulations between the two ventilator weaning paths during hospitalization. Tracheostomy decannulation is an established marker of the resolution of severe hypoxemic respiratory failure(30). At the completion of the study we will be able assess if one or other weaning path is superior in providing sustained resolution of the respiratory failure.
- Scientific aim 3. In this exploratory aim we will evaluate if time to speaking valve use, intensive care unit transfers, hospital length of stay, in-hospital and 90-day mortality and change in the Functional Status Score for the Intensive Care Unit (FSS-ICU) defers between the two weaning protocol paths.

3.0 BACKGROUND AND RATIONALE

Background

PMV is an important public health problem affecting 18,000 to 39,000 people in the USA(2, 14). PMV is defined by patients requiring at least 6 hours of positive pressure MV beyond 21 days(2), but it encompasses a wide variety of disease severity with significant outcome differences. Following acute care hospitalization patients with continued MV need are often transferred to LTACHs for continued ventilator weaning(27) and they represent the most severely ill PMV patients. The most common factors that challenge PMV weaning in the LTACH are: 1. poor baseline functional status, 2. severe course of underlying acute illness, 3. hemodynamic instability 4. variable expertise in mechanical ventilator weaning by treatment team and 5. the need for complex long term care. Most PMV patients in the LTACH have a tracheostomy tube for chronic mechanical ventilation which further complicates weaning(31). Because of the complexity of care, outcomes of ventilator weaning in the LTACH are poor and most patients die within 1 year(4, 9). PMV weaning success is often measured by discontinuation of mechanical ventilation, but tracheostomy decannulation is probably the more important event, which signifies the resolution of severe respiratory failure. It is estimated that only about 35-59% of PMV patients are decannulated at the LTACH, but this is based on small observational studies and the true success rate of decannulation is not known(32, 33).

Significance

While MV weaning has been extensively studied in acute care hospital intensive care units(11) there is lack of strong scientific evidence how to apply this knowledge to the PMV patients. Jubran et al. studied PSV weaning in LTACH patients and compared their success rate to unassisted breathing trials. This study focused on patients who were able to sustain unassisted breathing measured with spontaneous breathing trial (SBT).(3) Unfortunately, the majority of PMV patients who arrive for ventilator weaning to the LTACH cannot sustain unassisted breaths and fail SBT. In clinical practice at our institution(10) and elsewhere,(9) these patients participate in TIPS or PSV weaning programs. Other ventilator weaning modalities have been considered largely experimental due to technical and safety concerns in this patient population(6). Despite the common use of these ventilator weaning programs, they have not been compared for weaning and tracheostomy decannulation success. Currently, it is purely the clinician’s decision which method is chosen. We believe by studying the success of the ventilator programs in a clinical trial we will be able to learn about their benefits and also critically evaluate the challenges that hinder the overall ventilator weaning success.

Brief description of weaning programs

1. TIPS weaning. This ventilator weaning program starts with SIMV ventilation with a set respiratory rate of 10 to allow close evaluation of hemodynamic stability. The mandatory respiratory rate is then reduced daily for 4 days to allow assessment of spontaneous rate. If the patient remains hemodynamically stable, the weaning program continues with PSV weaning performed for 5 days while reducing the pressure support daily. If a patient is able to able to tolerate low pressure support, the patient is disconnected from the ventilator daily for increasing periods of time. The minimum length of the TIPS weaning program is 21 days. While this program is longer, in theory, it allows the patient more sustained stability when off the ventilator. The program is detailed in Table 2.
2. PSV weaning. In the PSV weaning program, patients receive PSV ventilation for 12 hours daily then rest on the original mandatory ventilator settings. The pressure support is reduced daily for 6 consecutive days. If a patient is able to tolerate low pressure support, the patient is disconnected from the ventilator daily for increasing periods of time. The minimum length of the PSV weaning program is 14 days. This program follows the philosophy that daily aggressive weaning combined with nighttime rest results in sustained ventilator-free time. The program is detailed in Table 3.

Both protocols end if the patient is able to tolerate at least 72 hours of disconnect from the ventilator.

4.0 OVERVIEW OF STUDY DESIGN/INTERVENTION

4.1 Design:

To study the non-inferiority of the PSV path compared to the TIPS path in ventilator weaning, consented patients will be randomized in two groups after admission to BRH. Both groups will undergo a daily weaning protocol performed by a respiratory therapist. The program allows daily assessment for respiratory and hemodynamic function.

To achieve our primary aim we will assess the success rate of MV weaning rate in both groups daily. Weaning failure will be defined as hemodynamic or respiratory instability, which would prevent them with continuing with the weaning program.

To achieve our secondary aim, we will compare the in hospital tracheostomy decannulation rate between the two ventilator weaning groups.

To achieve our third aim, we will collect demographic, clinical, and hospital information. We will extract pre-admission data from available short-stay acute care hospitals (STACH) notes and collect data prospectively from the BRH electronic medical records (EMR). The list of collected parameters is shown in Table 4 in Section 7. The data will be used to generate hospital outcomes which will be compared between the two groups of patients. The list of secondary outcomes is shown in Table 1. These hospital outcomes have been previously used in clinical studies to assess ventilator weaning success(4, 21).

Table 1. List of hospital outcomes

| 1. time to speaking valve use (days) |
| --- |
| 1. intensive care unit transfer (yes/no) |
| 1. hospital length of stay (LOS, days) |
| 1. in-patient mortality (yes/no) |
| 1. 90-day mortality (yes/no) |
| 1. Functional Status Score for the Intensive Care Unit (FSS-ICU) score on admission and at discharge |

4.2 Intervention

**A.** **Study protocol** (Figure 1):

1. Patients will be considered for enrollment in the study within 72 hours of admission to BRH.
2. All BRH patients requiring positive pressure invasive mechanical ventilation (MV) undergo spontaneous breathing trial (SBT, pressure support ventilation (PS) 5cmH2O with 5cmH2O of positive end expiratory pressure, PEEP) per hospital policy within 24 hours of admission. If they pass the SBT, they will be eligible for cool aerosol trials (humidified, oxygenated air without positive pressure mechanical ventilation). These patients will be excluded from the study, except if they fail to tolerate cool aerosol within 24 hours and continue require continuous positive pressure ventilation. These patients can be considered for study enrollment the next day.
3. In the rare instance that a patient has an endotracheal tube on admission to BRH and continued MV is needed, a date for tracheostomy will be set. Patients following tracheostomy placement can participate in the study.
4. Parameters to preclude study enrollment are listed in the Exclusion criteria (Section 5.2).
5. All patients are admitted to monitored beds at BRH. Vital signs (heart rate, oxygen saturation and lead 2 electrocardiogram, respiratory rate and ventilator compliance) are continuously monitored. Blood pressure is measured every 6 hours. Hemodynamic and respiratory stability is assessed daily on these parameters by the nurse and the respiratory therapist. The list of hemodynamic and respiratory parameters with values that represent instability are listed in section 5.3.
6. On D0, if a patient is stable and required continued MV, the study team will approach the patient and/or the patient’s designated power of attorney (DPOA) for informed consent.
7. On D0, consented patients, now referred to as subjects, will be randomized to participate in the fast or slow path of ventilator weaning.
8. On D1, weaning starts. Hemodynamic and respiratory instability is assessed every day as in point 5. In unstable subjects the study is halted, but they will be followed throughout their hospitalization at BRH for hospital outcomes. Weaning can be attempted after stabilization per the decision of the attending pulmonologist by restarting the same path from step 1.
9. If a subject fails to progress to the next step of ventilator weaning, the same step can be repeated on 3 consecutive days and if possible, he/she can continue with the designated weaning path.
10. If a subject cannot restart the weaning within 30 days, their participation in the study is considered complete.
11. Both paths will consist of a MV and a cool aerosol (CA) trial parts. The length of the TIPS path is a minimum 21 steps and 14 steps for the PSV path. These usually refer to days, but subjects may progress up to 3 steps a day. The details of both path are shown in the “ventilator paths” section.
12. When a subject is transitioned from MV to CA, arterial blood gas (ABG), will be collected after 2hrs CA to ensure adequate oxygenation and to avoid elevated carbon dioxide levels.
13. Following completion of the study all subjects will be assessed for tracheostomy decannulation by pulmonology, speech pathology and respiratory therapy.


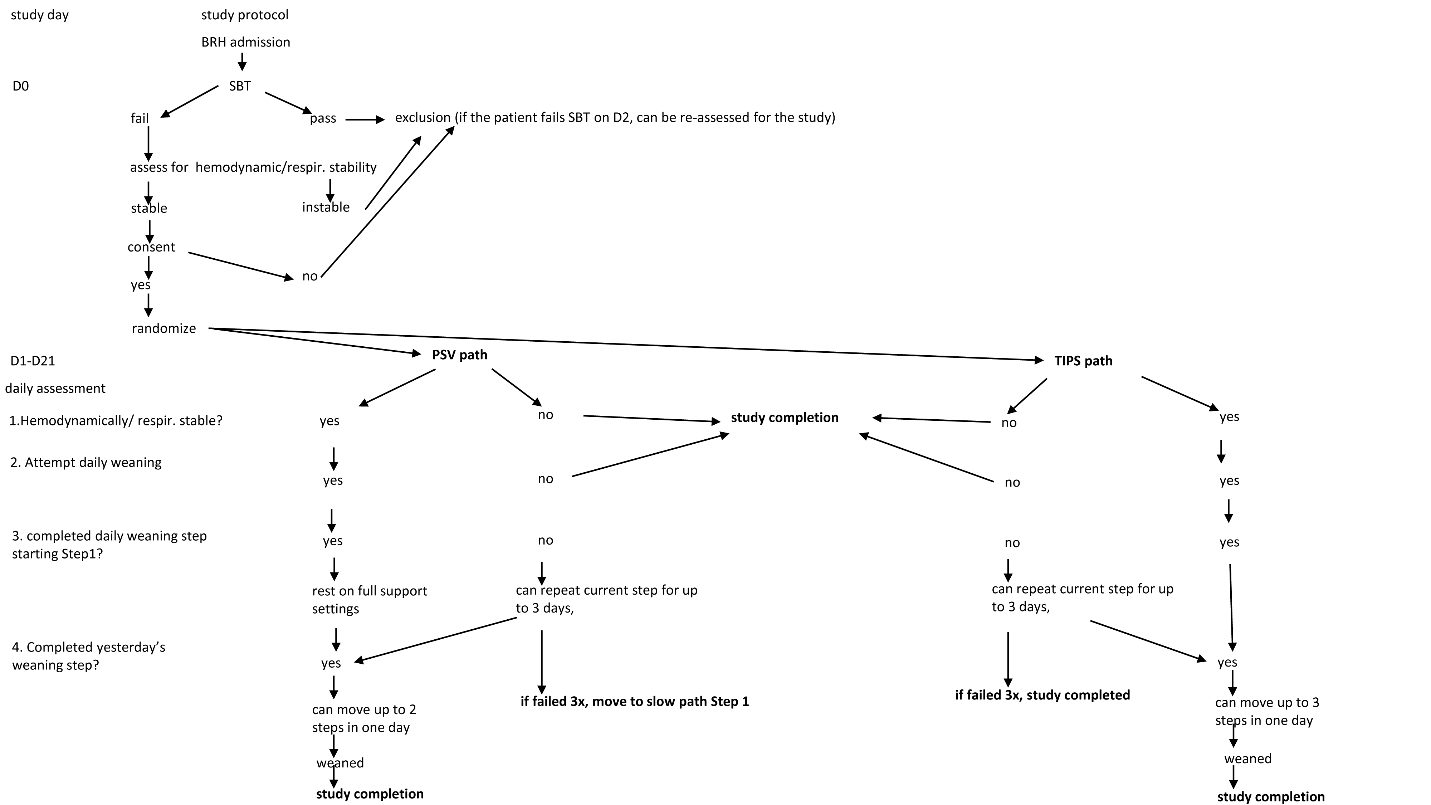
Figure 1

**B. Ventilator paths**

1. TIPS path (Table 2). In the TIPS path, subjects will complete a 21-step protocol. MV weaning is done with synchronized intermittent mandatory ventilation (SIMV) mode for 24 hrs. Patients will transition to this mode on the D1 after completion of SBT. The initial ventilator setup is PS 20 cmH2O PEEP 5cmH2O set RR 10/min. During the first 4 steps the backup rate will be lowered gradually from 8/min to 4/min (Step 1-4). Subsequently the PS will be reduced from 20 to 10 by each step (Step 5-9). SBT is performed in Step 10 with 1 hr. CA trials. ABG is performed in Step 11 with 2 hrs. of CA to assure respiratory stability. Respiratory stability is defined by pH > 7.30, paO2>55mmHg, pCO2<60 mmHg or no more than 10 mmHg change from admission baseline pCO2. This protocol has a slower increase in CA time slowly to allow observation for stability and reaches 24 hours by Step 19. Patients rest on SIMV PS=10 cmH2O PEEP=5cmgH2O, RR=4 (Step 9) setting during CA trial. Subjects can progress up to 3 steps daily, if deemed able by the respiratory therapist. The protocol completes when the patients is able to complete 72 hours of continuous CA time.

Table 2. TIPS path protocol.

| Step 1. Transition from admission ventilator mode to SIMV PS=20 cmH2O PEEP=5cmH2O, RR=10 |
| --- |
| Step 2. SIMV PS=20 cmH2O PEEP=5cmH2O RR=8 |
| Step 3. SIMV PS=20 cmH2O PEEP=5cm H2O RR=6 |
| Step 4. SIMV PS=20 cmH2O PEEP=5cm H2O RR=4 |
| Step 5. SIMV PS=18 cmH2O PEEP=5cm H2O RR=4 |
| Step 6. SIMV PS=16 cmH2O PEEP=5cm H2O RR=4 |
| Step 7. SIMV PS=14 cmH2O PEEP=5cm H2O RR=4 |
| Step 8. SIMV PS=12 cmH2O PEEP=5cm H2O RR=4 |
| Step 9. SIMV PS=10 cmH2O PEEP=5cm H2O RR=4 |
| Step 10. 1 hrs. CA rest on SIMV PS=10 cmH2O PEEP=5cm H2O RR=4 |
| Step 11. 2 hrs. CA rest on SIMV PS=10 cmH2O PEEP=5cm H2O RR=4. ABG is performed |
| Step 12. 4 hrs. CA rest on SIMV PS=10 cmH2O PEEP=5cm H2O RR=4 |
| Step 13. 6 hrs. CA rest on SIMV PS=10 cmH2O PEEP=5cm H2O RR=4 |
| Step 14. 8 hrs. CA rest on SIMV PS=10 cmH2O PEEP=5cm H2O RR=4 |
| Step 15. 10 hrs. CA rest on SIMV PS=10 cmH2O PEEP=5cm H2O RR=4 |
| Step 16. 12 hrs. CA rest on SIMV PS=10 cmH2O PEEP=5cm H2O RR=4 |
| Step 17. 16 hrs. CA rest on SIMV PS=10 cmH2O PEEP=5cm H2O RR=4 |
| Step 18. 20 hrs. CA rest on SIMV PS=10 cmH2O PEEP=5cm H2O RR=4 |
| Step 19. 24 hrs. CA |
| Step 20. 48 hrs. CA |
| Step 21. 72 hrs. CA- protocol completed |

Abbreviations: SIMV=synchronized intermittent mandatory ventilation, RR= respiratory rate, PS=pressure support, PEEP=positive end expiratory pressure, SBT= spontaneous breathing trial, ABG arterial blood gas, CA=cool aerosol. The same abbreviations are used in the subsequent tables.

1. PSV path (Table 3). In the PSV path subjects will complete a 14 step protocol. In this protocol gradual decrease of PS is used for 10-12 hrs. a day. PS is decreased from 20cm H2O to 10cmH2O with 5cmH2O PEEP applied (Step 1-6). Every weaning step is followed by rest on the same ventilator setting the patient had on admission. Starting Step 7, subjects will undergo daily SBT (1 hour 5cmH2O PS with 5cmH2O PEEP). If they pass, they will progress to CA trials (Step 8-14). ABG is performed after 2 hours of CA time in Step 7 to assure respiratory stability. Respiratory stability is defined by pH > 7.30, paO2>55mmHg, pCO2<60 mmHg or no more than 10 mmHg change from admission baseline pCO2. CA time will be extended by 4 hrs. in Step 8 to 14. Patients rest on the admission ventilator settings. Subjects can progress up to 3 steps daily, if deemed able by the respiratory therapist. The protocol completes when the patients is able to complete 72 hrs. of continuous CA time.

Table 3. PSV path protocol

| Step1. PS=20cmH2O PEEP=cm5H2O Backup rate=10* |
| --- |
| Step 2. PS=18 cmH2O PEEP=5 cmH2O Backup rate=10 |
| Step 3. PS=16 cmH2O PEEP=5 cmH2O Backup rate=10 |
| Step 4. PS=14 cmH2O PEEP=5 cmH2O Backup rate=10 |
| Step 5. PS=12 cmH2O PEEP=5 cmH2O Backup rate=10 |
| Step 6. PS=10 cmH2O PEEP=5 cmH2O Backup rate=10 |
| Step 7. SBT, if passes, oxygenated Cool aerosol (CA) for 4 hrs. (keep O2 sat>90%), Perform ABG after 2 hrs.# |
| Step 8. SBT, if passes, CA for 8 hrs. |
| Step 9. SBT, if passes, CA for 12 hrs. |
| Step 10. SBT, if passes, CA for 16 hrs. |
| Step 11. SBT, if passes, CA for 20 hrs. |
| Step 12. SBT, if passes, CA for 24 hrs. |
| Step 13. CA for 48hrs |
| Step 14. CA for 72 hrs-protocol completed |

* weaning protocol is performed for 10-12 hrs. daily and subjects rest on admission ventilator settings. # subjects rest on admission ventilator settings during CA trial.

5.0 CRITERIA FOR SUBJECT ELIGIBILITY

The study population consist of patients admitted to BRH main site for ventilator weaning. BRH main site is a 50-bed LTACH in Los Angeles, which specializes in ventilator weaning. Patients arrive to BRH from short-term acute care hospitals (STACH) in the greater Los Angeles area. Patients who required prolonged continuous positive pressure ventilation (greater than 21 days, PMV) will be invited to participate in the study. The majority of patients arrive with tracheostomy already placed for continued mechanical ventilation, but patients who arrive with endotracheal tube (ETT) can also participate as long as we can arrange for tracheostomy before the start of the study.

5.1 Subject Inclusion Criteria

1. Patients requiring positive pressure mechanical ventilation for at least 21 days prior to BRH admission and
2. Have a secure tracheostomy.

5.2 Subject Exclusion Criteria

1. Inability to obtain informed consent from patient or DPOA
2. Incarcerated patients
3. Patients with less than 3 months of life expectancy
4. Patients requiring vasopressor medication to stabilize blood pressure on admission
5. Systolic blood pressure less than 90mmHg on admission
6. Pulse less than 50 or greater than 130 beats per minute or change by more than 20 from baseline on admission
7. Respiratory rate greater than 35/min
8. Oxygen saturation less than 90%
9. PEEP>5cmH2O
10. Lung tidal volume less than 250ml despite MV support
11. At least one previous admission to BRH with unsuccessful ventilator liberation attempt
12. Length of Stay (LOS) at BRH less than 24hours
13. Patients pass spontaneous breathing trial (SBT) on D1 and eligible for cool aerosol, except if they fail SBT on D2 and require continued MV

5.3 Daily assessment for hemodynamic and respiratory stability

1. Patients requiring vasopressor medication to stabilize blood pressure
2. Systolic blood pressure less than 90mmHg
3. Pulse less than 50 or greater than 130 beats per minute or change by more than 20 change from baseline
4. Respiratory rate greater than 35/min on current MV settings
5. Oxygen saturation less than 90%
6. Lung tidal volume less than 250ml despite MV support

6.0 RECRUITMENT PLAN

All patients with PMV will be considered for study participation regardless of age, gender, race or ethnicity as long as informed consent can be obtained. There is no incentive to participate other than the results of this study may help to move forward the field of PMV and will benefit patients in the future. There is a sizable incarcerated population at BRH. We will exclude incarcerated patients because they and their DPOA will not be able to consent freely to the study.

On D0, patients and/or their DPOA will be approached for informed consent. Our intent is to recruit 300 patients with 150 patients in each path of the study.

**7.0 STATISTICAL CONSIDERATIONS**

7.1 Statistics

Primary analyses will be by intent to treat, but a secondary per protocol analysis will also be performed.

For the primary outcome, the proportion who are successfully weaned before 30 days on their original protocol, we will use Fisher’ exact test to carry out a non-inferiority test with a non-inferiority difference (Δ) of 15%. That is, we will test the null hypothesis that outcome under the PSV protocol is 15% or more worse than the outcome under TIPS protocol versus the one sided alternative that the outcome under PSV protocol is less than 15% worse.

Fisher’s exact test will also be used for comparing other secondary binary outcomes including tracheostomy decannulation (secondary aim), ICU transfer, inpatient mortality and 90-day mortality), using the usual null hypothesis of no difference. Proportions, differences in proportions and their corresponding 95% confidence bounds will be reported.

We will use the Kaplan-Meir method to compute time to event curves for time dependent hospital outcomes such as time to speaking valve use, time to successful completion of the weaning and time to transition from PSV to TIPS path. We will use the log rank test to compute p-values for comparing time to event curves. We will report full descriptive statistics by group (minimum, quartiles, mean, SD) for continuous outcomes such as hospital length of stay and FSS-ICU change and use the non-parametric Wilcoxon rank sum test to compute p-values since these outcomes do not have a normal distribution.

For covariate comparisons and adjustment we will report descriptive statistics by protocol group for age, gender, race/ethnicity, and baseline heart rate, respiratory rate, systolic blood pressure, diastolic blood pressure, oxygen saturation, ventilator tidal volume (Vt) and FSS-ICU to demonstrate that the randomization was successful, as expected. If needed we will carry out covariate adjusted comparisons using inverse propensity score weighting to carry out the adjustment and report both adjusted and unadjusted results. The propensity score, if needed, will be estimated using logistic regression using the baseline covariates above as predictors.

While we do not expect substantial dropouts or missing data, if there are dropouts or missing, we will use Cox proportional hazard regression to determine if time to dropping out is related to treatment group (TIPS or PSV), and baseline variables above. If there is no association, we will assume this is evidence for the dropouts to be at random (missing completely at random –MCAR). In this case, the methods above are known to give unbiased results. Otherwise, we will consider using multiple imputation if needed and compare estimates under multiple imputation versus estimates with complete data to see if this has a significant effect on the results

7.2 Sample size / power

The sample size is based on the primary outcome, the proportion successfully weaned on the original protocol by 24 days and is based on one sided non inferiority testing with a delta of 15%. From the study of Scheinhorn et al (6), we expect the success proportion in the TIPS group to be approximately 60%. Based on this, and assuming that there is no true difference between PSV and TIPS paths, a sample size of n=131 per group gives 80% power using a conservative two sided alpha=0.05 (one sided alpha=0.10). Therefore, our sample size of 150 per group should provide more than 80% power.

8.0 ASSESSMENT/EVALUATION PLAN

We will collect 4 groups of data from subjects. The detailed list is shown in Table 4. Study visit data collection overview is shown in the Appendix.

1. Preadmission and admission data will be collected for consented subjects on D0. Data will extracted from available STACH charts the BRH EMR by the study staff. Collected parameters are listed in Table 4, Column A and B.
2. Daily assessment data will be collected from D1 to discharge. Daily assessment parameters will be extracted from BRH EMR by the study staff. Collected parameters are listed in Table 4, Column C.
3. Discharge data will be collected upon discharge from BRH EMR by the study staff. Collected parameters are listed in Table 4, Column D.
4. Functional Status Score for the Intensive Care Unit (FSS-ICU) score will be calculated by the physical therapist on admission and at discharge. The data will be entered in the BRH EMR and collected by the study staff. The FSS-ICU score measures five basic abilities: a) the ability of rolling, b) transferring from lying to sitting, c) sitting at the edge of bed, d) transfer from sit to stand, and d) walking. Each task is scored on a 0 (no function) to 7 (independent performance) scale. The minimum combined score is 0 and the maximum is 35. The FSS-ICU score has been validated in both the ICU and the LTACH settings. Patients were evaluated by a physical therapist on admission and discharge, and the change in FSS-ICU score was used to access for functional change.
5. Tracheostomy decannulation evaluation. Decannulation will be discussed by the clinical team upon successful ventilator weaning and at least 72 hours of clinical stability on CA. The clinical team usually includes the speech pathologist, respiratory therapist and pulmonologist. The evaluation results will be entered in the BRH EMR and collected by the staff.
6. The 90-day post discharge mortality will be extracted from National Death Records.

Table 4. Data collection

| 1. **Preadmission data** | 1. **Admission data** | 1. **Daily assessment** | 1. **Discharge data** |
| --- | --- | --- | --- |
| **Demographic data** | admission date | hemodynamically stable (SBP>90, HR>50 and <120, RR>10 and <35 | discharge date |
| age | Hemodialysis need on admission | Assigned path: fast or slow? | Trach present (yes or no)? |
| gender | Admission Functional Status Score for the Intensive Care Unit (FSS-ICU) score | Study day? | Invasive mechanical ventilation (MV, yes or no)? |
| race | Endotracheal tube present | Study step? | If MV, mode, FiO2, RR, PEEP |
| ethnicity | Tracheostomy present | Okay to proceed to next step? | Discharge location: home, acute inpatient rehab (ARU) , skilled nursing facility (SNF), STACH readmission, dead) |
| **Chronic premorbid conditions (stable)** | hemodynamically stable (SBP>90, HR>60 and <120, RR>10 and <40 | Weaning failed (day) | If STACH readmission, provide reason |
| diabetes | Glasgow coma scale (GCS) | Study completion (day) | Hemodialysis on discharge (yes or no) |
| hypertension (HTN) | ventilator mode (pressure support, pressure control, volume control, synchronized intermittent mandatory ventilation) | Ventilator settings on the day of failure (mode, FiO2, RR, PEEP | FSS-ICU score on discharge |
| cerebrovascular accident (CVA) | FiO2 | Vt | BUN |
| coronary artery disease (CAD) | ventilator set rate | Oxygen saturation (O2sat) | Cr |
| chronic kidney disease (CKD) | spontaneous breathing rate | If fast path, moved to slow? | Plt |
| chronic hemodialysis (HD) | PEEP | If fast path and moved to slow, move date? |  |
| congestive heart failure (CHF) | Vt | Passy-Muir (speaking) valve use? |  |
| chronic obstructive lung disease (COPD) | SBP | Passy-Muir (speaking) valve time (hrs.) |  |
| pulmonary fibrosis (PF) | DBP | trach size |  |
| obesity (BMI>35) | HR | trach change (date) |  |
| neuromuscular disease | ABG | Swallow evaluation (date) |  |
| chronic malignancy | Cr | Swallow evaluation (pass or fail) |  |
| **acute premorbid condition on short-term acute care hospital (STACH) admission** | BUN | eligible for decannulation (date) |  |
| STACH admission date | Plt | decannulated (date) |  |
| cardiac arrest | Passy-Muir (speaking valve) use | Inpatient mortality (date) |  |
| vasopressor need | Feeding tube use | bronchoscopy need (yes or no) |  |
| sepsis |  | bronchoscopy date |  |
| acute kidney injury (AKI) |  | bronchoscopy reason |  |
| acute CVA |  | Intensive care unit (ICU) transfer |  |
| acute myocardial infarction (AMI) |  | ICU transfer date |  |
| acute hypoxemic respiratory failure |  | ICU transfer reason |  |
| pneumonia |  | cardiac arrest at BRH? |  |
| acute respiratory distress syndrome (ARDS) |  | Cardiac arrest date? |  |
| acute venous thromboembolism (VTE including deep venous thrombosis and pulmonary embolism) |  | vasopressor use |  |
| acute traumatic brain injury (TBI) |  | vasopressors start date |  |
| acute malignancy |  | new antibiotic use |  |
| acute gastrointestinal bleed |  | new antibiotic start date |  |
| acute liver failure |  | ICU discharge date |  |
| **preadmission respiratory condition** |  | ABG (during cool aerosol trial) |  |
| date of endotracheal intubation |  | SBP |  |
| date of tracheostomy |  | DBP |  |
| previous tracheostomy (yes or no)? |  | HR |  |
| **Laboratory data on STACH discharge** |  |  |  |
| Cr |  |  |  |
| BUN |  |  |  |
| PLT |  |  |  |

Abbreviations of vital signs and laboratory test no listed in previous tables: HR=heart rate, SBP= systolic blood pressure (mmHg), DPB=diastolic blood pressure (mmHg), O2 sat=oxygen saturation (%), FiO2=fractional inspired oxygen (%), Vt=ventilator tidal volume (ml), Cr=serum creatinine (mg/dl), BUN=serum blood urea nitrogen (mg/dl), Plt=platelet count (/mm3)

**9.0 ADVERSE EVENTS, SAFETY AND MONITORING**

There are four potential risk for adverse events:

1. Clinical deterioration. The study population consists of chronically critically ill patients with high risk of cardiopulmonary decompensation and death. Data from our team shows that inpatient mortality in the PMV population is approximately 14% (18). To protect the patient population, all BRH patients are continuously be monitored via telemetry for HR, O2 saturation and 1-lead EKG. Blood pressure is measured at least every 6 hours and as needed. Patients are followed by nurses and respiratory therapists 24 hours a day. These same rules apply to our study population. In the event of cardiopulmonary decompensation, the rapid response team or the physician on site is notified. BRH is staffed with onsite critical care trained physicians 24 hours a day. BRH also has a medical ICU for critical care. For determination of failed weaning, see Determination of failed weaning session.
2. Confidentiality. To avoid unauthorized access to study data: A. All personal and clinical data will be stored in a password protected database. B. Subjects will be assigned a code for identification which will be stored separately from the collected personal information. In case of breach of confidentiality the subjects and their DPOA will be notified and the study will halt.
3. Voluntary participation. Patients and/or their DPOA will be approached for consent to participate in the study. Only
4. Adverse events related to the study protocol. In case of hemodynamic and respiratory instability related to the ventilator protocol, the respiratory therapist will stop the weaning and the patient will be returned to the last safe ventilator setting.

To assure appropriate oversight the study team will contract with an Independent Safety Monitor Dr. Caleb Hsieh. The clinical trial will also be listed at the clinicaltirals.gov website.

## **10. ASSESSMENT OF SAFETY**

## 10.1 Determination of failed weaning

To adequately assess patient well-being and avoid major side effects from mechanical ventilation weaning, criteria for early study completion were developed. The criteria listed will be continuously monitored throughout the study by hospital staff. If a study subject meets one of the criteria listed below on 3 consecutive days, the subject will be excluded from the trial. However after exclusion from the trial the subject will be followed by the study staff for the remaining hospitalization time at BRH to collect clinical information shown in Table 4.

1. Hemodynamic instability during ventilator weaning.
2. Patients requiring vasopressor medication to stabilize blood pressure
3. Systolic blood pressure less than 80mmHg
4. Pulse less than 50 or greater than 130 beats per minute or change by more than 20 from baseline on admission
5. Respiratory instability during ventilator weaning.
6. Respiratory rate greater than 35/min
7. Oxygen saturation less than 90%
8. PEEP>5cmH2O
9. Lung tidal volume less than 250ml despite MV support
10. Ventilator-patient dyssynchrony resulting in more than 2 discordant breaths per 10 seconds despite adjustment of ventilator pressure, flow or volume settings
11. On ABG, pH<7.30, paO2<55mmHg, pCO2 >60 mmHg or no more than 10 mmHg change from admission baseline pCO2.
12. New onset fever, identified as core temperature >101F
13. Acute gastrointestinal bleed, identified as melena or bright blood per rectum.
14. Agitation, defined as pulling on tubes and line risking self-harm despite soft restraints.
15. Significant decline in cognition defined by unresponsiveness to command, if the patient was previously responsive.
16. Significant airway bleed resulting in suctioning difficulty of the airway

If a patients meets one of the failed weaning criteria, the respiratory therapist will return the patient to full support ventilator support, alert the attending pulmonologist and a member of the study team.

# 10.2 Unanticipated Problems

The Office for Human Research Protection Program (OHRPP) considers unanticipated problems involving risks to subjects or others to include, in general, any incident, experience, or outcome that meets **all** of the following criteria:

Unexpected in terms of nature, severity, or frequency given (1) the research procedures that are described in the protocol-related documents, such as the IRB-approved research protocol and informed consent document; and (2) the characteristics of the subject population being studied; related or possibly related to participation in the research (“possibly related” means there is a reasonable possibility that the incident, experience, or outcome may have been caused by the procedures involved in the research); and suggests that the research places subjects or others at a greater risk of harm (including physical, psychological, economic, or social harm) than was previously known or recognized.

Given that the study population has by definition severely deranged physiology and laboratory

values and a high risk of death as a fundamental part of their underlying disease process,

abnormal laboratory values and deaths are not considered unanticipated problems for this

population. Rather, we will be tracking physiological parameters listed here and in Section 10.1 to identify deterioration possibly related to the study protocol:

1. Hemodynamic stability
2. Respiratory stability
3. New onset fever
4. Acute gastrointestinal bleed
5. Agitation
6. Decline in cognition
7. Significant airway bleeding

#### 10.3 Adverse Events

An adverse event (AE) is any untoward or unfavorable medical occurrence in a human subject, including any abnormal sign temporally associated with the subject’s participation in the research, whether or not considered related to the subject’s participation in the research.

#### 10.4 Serious Adverse Events

A serious adverse event (SAE) is one that meets one or more of the following criteria:

- Results in death
- Is life-threatening (places the subject at immediate risk of death from the event as it occurred)
- Results in intensive care unit hospitalization or prolongation of existing hospitalization
- Results in a persistent or significant disability or incapacity

An important medical event that may not result in death, be life threatening, or require hospitalization may be considered an SAE when, based upon appropriate medical judgment, the event may jeopardize the subject and may require medical or surgical intervention to prevent one of the outcomes listed in this definition.

#### 10.5 Time Period and Frequency for Event Assessment and Follow-Up

Unanticipated problems will be recorded in the data collection system throughout the study.

The PI will record all reportable events with start dates occurring any time after enrollment until 7 days later for non-serious AEs, and 30 days for SAEs. Events will be followed for outcome information until resolution or stabilization.

#### 10.6 Characteristics of an Adverse Event and Relationship to Study Intervention

To assess relationship of an event to study intervention, the following guidelines are used:

- - 1. Related (Possible, Probable, Definite)
       1. The event is known to occur with the study intervention.
       2. There is a temporal relationship between the intervention and event onset.
       3. The event abates when the intervention is discontinued.
       4. The event reappears upon a re-challenge with the intervention.
    2. Not Related (Unlikely, Not Related)

1. There is no temporal relationship between the intervention and event onset.
2. An alternate etiology has been established

#### 10.7 Expectedness of SAEs

The Study PI will be responsible for determining whether an SAE is expected or unexpected. An adverse event will be considered unexpected if the nature, severity, or frequency of the event is not consistent with the risk information previously described for the intervention.

#### 10.8 Severity of Event

The following scale will be used to grade adverse events:

- - 1. Mild: no intervention required; no impact on activities of daily living (ADL)
    2. Moderate: minimal, local, or non-invasive intervention indicated; moderate impact on ADL
    3. Severe: significant symptoms requiring invasive intervention; subject seeks medical attention, needs major assistance with ADL

#### 10.9 Unanticipated Problem Reporting to IRB

Incidents or events that meet the OHRP criteria for unanticipated problems require the creation and completion of an unanticipated problem report form. OHRP recommends that investigators include the following information when reporting an adverse event, or any other incident, experience, or outcome as an unanticipated problem to the IRB:

- - - appropriate identifying information for the research protocol, such as the title, investigator’s name, and the IRB project number;
    - a detailed description of the adverse event, incident, experience, or outcome;
    - an explanation of the basis for determining that the adverse event, incident, experience, or outcome represents an unanticipated problem;
    - a description of any changes to the protocol or other corrective actions that have been taken or are proposed in response to the unanticipated problem.

To satisfy the requirement for prompt reporting, unanticipated problems will be reported using the following timeline:

- - - Unanticipated problems that are serious adverse events will be reported to the IRB within 1 week of the investigator becoming aware of the event.
    - Any other unanticipated problem will be reported to the IRB within 2 weeks of the investigator becoming aware of the problem.
    - All unanticipated problems should be reported to appropriate institutional officials (as required by an institution’s written reporting procedures), the supporting agency head (or designee), and OHRP within one month of the IRB’s receipt of the report of the problem from the investigator.

#### 10.10 Reporting of Serious Adverse Event

The study clinician will complete a Serious Adverse Event Form and submit via fax or email within the following timelines:

- - - Serious adverse events regardless of relationship, will be reported by fax within 72 hours of site awareness.

All SAEs will be followed until resolution or stabilization.

#### 10.11 Halting Rules

Should any serious adverse events or repeated adverse events appear to be related to the study interventions, the PI will work with the IRB and Independent Safety Monitor Dr. Caleb Hsieh to determine the appropriateness of halting further enrollment in the study. Dr. Hsieh and the PI of this study do not have a formal mentor / mentee relationship or other arrangement that would preclude objectivity.

## **11. STUDY OVERSIGHT**

In addition to the PI’s responsibility for oversight, study oversight will be under the direction of an Independent Safety Monitor (ISM), Dr Caleb Hsieh, and performed on a quarterly basis. The ISM is independent of the study and will be available in real time to review and recommend appropriate action regarding adverse events and other safety

**12.0 REFERENCES**

1. Wunsch H, Linde-Zwirble WT, Angus DC, Hartman ME, Milbrandt EB, Kahn JM. The epidemiology of mechanical ventilation use in the United States. Crit Care Med. 2010;38(10):1947-53.

2. Lone NI, Walsh TS. Prolonged mechanical ventilation in critically ill patients: epidemiology, outcomes and modelling the potential cost consequences of establishing a regional weaning unit. Crit Care. 2011;15(2):R102.

3. Jubran A, Grant BJ, Duffner LA, Collins EG, Lanuza DM, Hoffman LA, et al. Effect of pressure support vs unassisted breathing through a tracheostomy collar on weaning duration in patients requiring prolonged mechanical ventilation: a randomized trial. JAMA. 2013;309(7):671-7.

4. Jubran A, Grant BJB, Duffner LA, Collins EG, Lanuza DM, Hoffman LA, et al. Long-Term Outcome after Prolonged Mechanical Ventilation. A Long-Term Acute-Care Hospital Study. Am J Respir Crit Care Med. 2019;199(12):1508-16.

5. Divo MJ, Murray S, Cortopassi F, Celli BR. Prolonged mechanical ventilation in Massachusetts: the 2006 prevalence survey. Respir Care. 2010;55(12):1693-8.

6. Boles JM, Bion J, Connors A, Herridge M, Marsh B, Melot C, et al. Weaning from mechanical ventilation. Eur Respir J. 2007;29(5):1033-56.

7. Dasta JF, McLaughlin TP, Mody SH, Piech CT. Daily cost of an intensive care unit day: the contribution of mechanical ventilation. Crit Care Med. 2005;33(6):1266-71.

8. Kaier K, Heister T, Wolff J, Wolkewitz M. Mechanical ventilation and the daily cost of ICU care. BMC Health Serv Res. 2020;20(1):267.

9. Scheinhorn DJ, Hassenpflug MS, Votto JJ, Chao DC, Epstein SK, Doig GS, et al. Post-ICU mechanical ventilation at 23 long-term care hospitals: a multicenter outcomes study. Chest. 2007;131(1):85-93.

10. Scheinhorn DJ, Chao DC, Stearn-Hassenpflug M, Wallace WA. Outcomes in post-ICU mechanical ventilation: a therapist-implemented weaning protocol. Chest. 2001;119(1):236-42.

11. Esteban A, Frutos F, Tobin MJ, Alia I, Solsona JF, Valverdu I, et al. A comparison of four methods of weaning patients from mechanical ventilation. Spanish Lung Failure Collaborative Group. N Engl J Med. 1995;332(6):345-50.

12. Perme C, Nawa RK, Winkelman C, Masud F. A tool to assess mobility status in critically ill patients: the Perme Intensive Care Unit Mobility Score. Methodist Debakey Cardiovasc J. 2014;10(1):41-9.

13. Damuth E, Mitchell JA, Bartock JL, Roberts BW, Trzeciak S. Long-term survival of critically ill patients treated with prolonged mechanical ventilation: a systematic review and meta-analysis. Lancet Respir Med. 2015;3(7):544-53.

14. Hill AD, Fowler RA, Burns KE, Rose L, Pinto RL, Scales DC. Long-Term Outcomes and Health Care Utilization after Prolonged Mechanical Ventilation. Ann Am Thorac Soc. 2017;14(3):355-62.

15. Gracey DR, Hardy DC, Koenig GE. The chronic ventilator-dependent unit: a lower-cost alternative to intensive care. Mayo Clin Proc. 2000;75(5):445-9.

16. Beduneau G, Pham T, Schortgen F, Piquilloud L, Zogheib E, Jonas M, et al. Epidemiology of Weaning Outcome according to a New Definition. The WIND Study. Am J Respir Crit Care Med. 2017;195(6):772-83.

17. Brochard L, Rauss A, Benito S, Conti G, Mancebo J, Rekik N, et al. Comparison of three methods of gradual withdrawal from ventilatory support during weaning from mechanical ventilation. Am J Respir Crit Care Med. 1994;150(4):896-903.

18. Weaver M, Goodin DA, Miller HA, Karmali D, Agarwal AA, Frieboes HB, et al. Prediction of prolonged mechanical ventilation in the intensive care unit via machine learning: a COVID-19 perspective. Sci Rep. 2024;14(1):30173.

19. Scheinhorn DJ, Hassenpflug M, Artinian BM, LaBree L, Catlin JL. Predictors of weaning after 6 weeks of mechanical ventilation. Chest. 1995;107(2):500-5.

20. Chang YC, Huang KT, Chen YM, Wang CC, Wang YH, Tseng CC, et al. Ventilator Dependence Risk Score for the Prediction of Prolonged Mechanical Ventilation in Patients Who Survive Sepsis/Septic Shock with Respiratory Failure. Sci Rep. 2018;8(1):5650.

21. Dolinay T, Jun D, Chen L, Gornbein J. Mechanical Ventilator Liberation of Patients With COVID-19 in Long-term Acute Care Hospital. Chest. 2022;161(6):1517-25.

22. Dubin R, Veith JM, Grippi MA, McPeake J, Harhay MO, Mikkelsen ME. Functional Outcomes, Goals, and Goal Attainment among Chronically Critically Ill Long-Term Acute Care Hospital Patients. Ann Am Thorac Soc. 2021;18(12):2041-8.

23. Kahn JM, Davis BS, Le TQ, Yabes JG, Chang CH, Angus DC. Variation in mortality rates after admission to long-term acute care hospitals for ventilator weaning. J Crit Care. 2018;46:6-12.

24. Newman H, Clunie G, Wallace S, Smith C, Martin D, Pattison N. What matters most to adults with a tracheostomy in ICU and the implications for clinical practice: a qualitative systematic review and metasynthesis. J Crit Care. 2022;72:154145.

25. Wood G, MacLeod B, Moffatt S. Weaning from mechanical ventilation: physician-directed vs a respiratory-therapist-directed protocol. Respir Care. 1995;40(3):219-24.

26. Kahn JM, Werner RM, David G, Ten Have TR, Benson NM, Asch DA. Effectiveness of long-term acute care hospitalization in elderly patients with chronic critical illness. Med Care. 2013;51(1):4-10.

27. Kahn JM, Benson NM, Appleby D, Carson SS, Iwashyna TJ. Long-term acute care hospital utilization after critical illness. JAMA. 2010;303(22):2253-9.

28. Makam AN, Nguyen OK, Miller ME, Shah SJ, Kapinos KA, Halm EA. Comparative effectiveness of long-term acute care hospital versus skilled nursing facility transfer. BMC Health Serv Res. 2020;20(1):1032.

29. Sumarsono N, Sudore RL, Smith AK, Pantilat SZ, Anderson WG, Makam AN. Availability of Palliative Care in Long-Term Acute Care Hospitals. J Am Med Dir Assoc. 2021;22(10):2207-11.

30. Stelfox HT, Crimi C, Berra L, Noto A, Schmidt U, Bigatello LM, et al. Determinants of tracheostomy decannulation: an international survey. Crit Care. 2008;12(1):R26.

31. Cox CE, Carson SS, Holmes GM, Howard A, Carey TS. Increase in tracheostomy for prolonged mechanical ventilation in North Carolina, 1993-2002. Crit Care Med. 2004;32(11):2219-26.

32. O'Connor HH, Kirby KJ, Terrin N, Hill NS, White AC. Decannulation following tracheostomy for prolonged mechanical ventilation. J Intensive Care Med. 2009;24(3):187-94.

33. Ghiani A, Tsitouras K, Paderewska J, Milger K, Walcher S, Weiffenbach M, et al. Incidence, causes, and predictors of unsuccessful decannulation following prolonged weaning. Ther Adv Chronic Dis. 2022;13:20406223221109655.

**10. APPENDIX** Study visit data collection overview

|  | **Visit 1 Day 0 of admission** | **Visit 2 Day 1-21 daily assessment** | **Visit 3 study completion** | **Visit 4 discharge** |
| --- | --- | --- | --- | --- |
| Informed Consent | **X** |  |  |  |
| Medical History (from previous hospital records) | **X** |  |  |  |
| Complete Physical Exam | **X** |  |  |  |
| Abbreviated Physical Exam |  | **X** | **X** | **X** |
| Height | **X** | **X** | **X** | **X** |
| Demographics | **X** |  |  |  |
| Weight | **X** | **X** | **X** | **X** |
| Vital Signs | **X** | **X** | **X** | **X** |
| Ventilator settings | **X** | **X** | **X** | **X** |
| O2 saturation | **X** | **X** | **X** | **X** |
| Ventilator liberation | **X** | **X** | **X** | **X** |
| Tracheostomy status | **X** | **X** | **X** | **X** |
| Laboratory data (BUN, Cr, Plt) | **X** | **X** | **X** | **X** |
| Randomization | **X** |  |  |  |
| Concomitant Medication Review (abx, vasopressors) | **X** | **X** | **X** | **X** |
| Adverse Experiences (ICU transfer, hemodynamical instability) |  | **X** |  |  |
| Death | **X** | **X** | **X** | **X** |

**UCLA IRB Informed consent**

**UNIVERSITY OF CALIFORNIA LOS ANGELES**

**CONSENT TO PARTICIPATE IN RESEARCH**

***Mechanical ventilator weaning in chronically ventilated patients***

**Lay Title: A clinical study comparing the safety and effectiveness of two commonly used ventilator weaning strategies**

**INTRODUCTION**

Tamas Dolinay, M.D., and associates from the Division of Pulmonary & Critical Care at the Department of Medicine at the University of California, Los Angeles are conducting a research study at Barlow Respiratory Hospital (BRH).

**KEY INFORMATION** :

The following is a short summary of this study to help you decide whether or not to be a part of this study. More detailed information is listed later on in this form.

**WHY AM I BEING INVITED TO TAKE PART IN A RESEARCH STUDY?**

We invite you to take part in a research study because you are over 18 years of age and are currently intubated, have a tracheostomy tube and undergoing mechanical ventilation with plans for weaning you off the ventilator in BRH. Weaning is the process of decreasing the degree of ventilator support and allowing you to assume a greater proportion of your own ventilation. Research studies are voluntary and include only people who choose to take part.

**WHAT SHOULD I KNOW ABOUT A RESEARCH STUDY?**

- Someone will explain this research study to you.
- Whether or not you take part is up to you.
- You can choose not to take part.
- You can agree to take part and later change your mind.
- Your decision will not be held against you.
- You can ask all the questions you want before you decide.
- You can discuss this study with friends and family.
- You can also discuss it with your health care doctor or request a second opinion.

**WHY IS THIS RESEARCH BEING DONE?**

Mechanical ventilation is a commonly used life-saving hospital procedure for patients with severe breathing difficulty. Some patients have difficulty separating from the ventilator and need to be removed gradually. This process is called ventilator weaning. It is not known what is the best way to wean patients from the ventilator. In our study, we will compare two commonly used ventilator weaning strategies and compare their success. One ventilator strategy, the Pressure Support Ventilation weaning (PSV), combines 12 hours ventilator weaning with 12 hours rest on the ventilator. This strategy is faster with an anticipated weaning in 2 weeks. The other strategy, the Therapistimplemented Patient Specific weaning (TIPS), gradually lowers support and weans in 3 weeks.

More detailed information about the study procedures can be found under ***“*WHAT**

**WILL HAPPEN IF I TAKE PART IN THIS STUDY?*”***

**WHAT KINDS OF RISKS OR DISCOMFORTS COULD I EXPECT?**

Clinical deterioration is one important risk in this study. The study population consists of chronically critically ill patients with high risk of cardiopulmonary decompensation (cessation of adequate heart and lung function) and death. All BRH patients are continuously monitored via telemetry (the automatic measurement and wireless transmission of data from remote sources) for heart rate, oxygen levels and electrocardiogram (ECG or EKG) measures the heart's electrical activity. Blood pressure is measured at least every 6 hours and as needed. Patients are followed by nurses and respiratory therapists 24 hours a day. These same rules apply to participants in our study. In the event of cardiopulmonary decompensation, the rapid response team or the physician on site is notified. BRH is staffed with onsite critical care trained physicians 24 hours a day. BRH also has a medical intensive care unit (ICU) for critical care.

More detailed information about the risks of this study can be found under ***“*WHAT**

**KINDS OF RISKS OR DISCOMFORTS COULD I EXPECT?  *(Detailed Description)”***

**ARE THERE ANY BENEFITS IF I PARTICIPATE?**

You will not directly benefit from your participation in the research. The benefits of the research may help others who are weaning off mechanical ventilation.

**WHAT OTHER CHOICES DO I HAVE IF I DON’T WANT TO PARTICIPATE?**

The only other alternative to participation is not to participate.

**HOW MANY PEOPLE WILL TAKE PART IN THIS STUDY?**

300 people will take part in this study at BRH.

**WHAT WILL HAPPEN IF I TAKE PART IN THIS STUDY?**

You will be asked to participate in this study within 72 hours of your admission to BRH.

You will undergo a spontaneous breathing trial (SBT) to assess your ability to breathe while receiving minimal or no ventilator support. If you pass the SBT, you will be eligible for cool aerosol trials (humidified, oxygenated air without positive pressure mechanical ventilation). If you fail SBT within 24 hours, you will be eligible for the study.

If you take part in this study, the researcher(s) will ask you to do the following:

**Day 0**

If you do meet all eligibility criteria, we will discuss the study participation details with you if your level of consciousness allows for it. In case you are not in the position to provide written consent, we will attempt to identify a health care proxy who is willing to make decisions about participation in the study on your behalf.

**Day 1 to Day 30**

We will look at your medical record and talk to your treatment team to confirm that you meet the criteria required to participate in the study. Ventilator weaning will be started. You will be randomized (assigned by chance, like the toss of a coin) to either PSV weaning or TIPS. PSV weaning combines 12 hours ventilator weaning with 12 hours rest on the ventilator. This strategy is faster with an anticipated weaning in 2 weeks. The other strategy, the TIPS, gradually lowers support and weans in 3 weeks. You will have an equal chance (50:50) of being assigned to either PSV or TIPS weaning. You will be monitored, and your condition will be assessed daily.

If you cannot be weaned off ventilator support, the respiratory therapist will return you to full support ventilator support, alert the attending pulmonologist and a member of the study team. The study doctor will determine if you have completed the study.

**WHAT KINDS OF RISKS OR DISCOMFORTS COULD I EXPECT? (Detailed Description)**

**Known risks and discomforts:**

Risks associated with ventilator weaning: Risks associated with ventilator weaning could differ between groups, e.g., overly rapid weaning could cause you to feel short of breath, develop problems with your heart rhythm, or not get enough oxygen to your heart - which could cause angina or a myocardial infarction (heart attack) or death. A slow method of weaning would mean that you are dependent on the ventilator for a longer period of time.

Risks associated with randomization: You will be assigned to a study group at random (by chance). Your assignment is based on chance (like a coin flip) rather than a medical decision made by the researchers. The study group you are assigned to might not be the group you would prefer to be in. It might also prove to be less effective or have more side effects than the other study groups(s), or standard treatments available for your condition.

Loss of confidentiality: As this study involves the use of your identifiable, personal information, there is a chance that a loss of confidentiality will occur. The researchers have procedures in place to lessen the possibility of this happening.

**Unknown risks and discomforts:**

The experimental treatments may have side effects that no one knows about yet. The researchers will let you know if they learn anything that might make you change your mind about participating in the study.

**HOW WILL INFORMATION ABOUT ME AND MY PARTICIPATION BE KEPT CONFIDENTIAL?**

The researchers will do their best to make sure that your private information is kept confidential. Information about you will be handled as confidentially as possible, but participating in research may involve a loss of privacy and the potential for a breach in confidentiality. Study data will be physically and electronically secured. As with any use of electronic means to store data, there is a risk of breach of data security.

**Use of personal information that can identify you:**

Any information that is obtained in connection with this study and that can identify you will remain confidential. It will be disclosed only with your permission or as required by law. Study data will be physically and electronically secured. With any use of electronic means to store data, there is a risk of breach of data security. The research team, authorized UCLA and BRH personnel and regulatory agencies may have access to study data and records to monitor the study. Research records provided to authorized, non- UCLA or BRH personnel will not contain identifiable information about you. Publications and/or presentations that result from this study will not identify you by name.

For the purposes of your participation in this study and to protect your identity, your study doctor will assign you a unique code, such as a series of numbers and/or letters. The study doctor will record the study data collected from you in a report form that uses your assigned code, not your name. This is to protect your study data by making it anonymous for most study purposes.

**How information about you will be stored:**

All data (clinical data and other records) kept at the site will be physically and electronically secured to maintain subject confidentiality. Paper records with subject data will be stored in locked office or cabinet. Computer records will be password protected, and encrypted when possible. The study database is maintained behind a secure firewall, access is password protected and uses encryption for all data entry and access. The full access to the data will be granted only to the study team members involved in data analysis.

**People and agencies that will have access to your information:**

The research team, authorized UCLA personnel, Barlow Respiratory Hospital staff, and regulatory agencies such as the Food and Drug Administration (FDA), may have access to study data and records to monitor the study. Research records provided to authorized, non-UCLA personnel will not contain identifiable information about you. Publications and/or presentations that result from this study will not identify you by name.

Employees of the University may have access to identifiable information as part of routine processing of your information, such as lab work or processing payment. However, University employees are bound by strict rules of confidentiality.

**How long information from the study will be kept:**

The researchers intend to keep the research data and records indefinitely.

**USE OF DATA AND SPECIMENS FOR FUTURE RESEARCH**

The researcher may decide to use the collected data without personal identifiers for future research.

**ARE THERE ANY COSTS FOR TAKING PART IN THIS STUDY?**

The study will pay for research-related items and/or services that are provided only because you are participating in the study. These research-related items and/or services are explained in other areas of this consent form.

You or your health plan may be responsible to pay for all the types of items listed below:

- Items and services that would have been provided to you even if you were not in the study
- Health care given during the study as part of your regular care
- Items or services needed to give you study drugs or devices
- Monitoring for side effects or other problems
- Deductibles or co-pays for these items and/or services

**WILL I BE PAID FOR MY PARTICIPATION?**

You will not be paid for your participation in this research study.

**WHO CAN I CONTACT IF I HAVE QUESTIONS ABOUT THIS STUDY?**

**The Research Team:**

If you have any questions, comments or concerns about the research, you can talk to the one of the researchers. Please contact Dr. Tamas Dolinay at (818) 260-8706 or 213-202-6844, or have the page operator page him 24 hours a day.

**UCLA Office of the Human Research Protection Program (OHRPP):**

If you have questions about your rights while taking part in this study, or you have concerns or suggestions and you want to talk to someone other than the researchers about the study, you may contact the UCLA OHRPP by phone: (310) 206-2040; by email: participants@research.ucla.edu or U.S. mail: UCLA OHRPP, Box 951406, Los Angeles, CA 90095-1406.

**WHAT HAPPENS IF I BELIEVE I AM INJURED BECAUSE I TOOK PART IN THIS STUDY?**

It is important that you promptly tell the researchers if you believe that you have been injured because of taking part in this study. You can tell the researcher in person or call him/her at the number listed above.

If you are injured as a result of being in this study, UCLA will provide necessary medical treatment. The costs of the treatment may be covered by the University of California or billed to you or your insurer just like other medical costs, depending on a number of factors. The University and the study sponsor do not normally provide any other form of compensation for injury. For more information about this, you may call the UCLA Office of the Human Research Protection Program at (310) 206-2040 or send an email to participants@research.ucla.edu.

**WHAT ARE MY RIGHTS IF I TAKE PART IN THIS STUDY?**

Taking part in this study is your choice. You can choose whether or not you want to participate. Whatever decision you make, there will be no penalty to you and you will not lose any of your regular benefits.

- You have a right to have all of your questions answered before deciding whether to take part.
- Your decision will not affect the medical care you receive from UCLA.
- If you decide to take part, you can leave the study at any time.
- If you decide to stop being in this study you should notify the research team right away. The researchers may ask you to complete some procedures in order to protect your safety.
- If you decide not to take part, you can still get medical care from UCLA.

**HOW DO I INDICATE MY AGREEMENT TO PARTICIPATE?**

If you want to participate in this study you should sign and date below. You have been given a copy of this consent form and the Research Participant’s Bill of Rights to keep. You will be asked to sign a separate form authorizing access, use, creation, or disclosure of health information about you.]

**SIGNATURE OF THE PARTICIPANT**

______________________________________

Name of Participant

______________________________________ ______________________

Signature of Participant Date

**SIGNATURE OF THE LEGALLY-AUTHORIZED REPRESENTATIVE**

______________________________________

Name of Legally-Authorized Representative

| ______________________________________ | ______________________ |
| --- | --- |
| Signature of Legally-Authorized Representative | Date |

**SIGNATURE OF PERSON OBTAINING CONSENT**

_____________________ ______________________

Name of Person Obtaining Consent Contact Number

______________________________________ ______________________

Signature of Person Obtaining Consent Date

**UNIVERSITY OF CALIFORNIA LOS ANGELES**

RESEARCH PARTICIPANT’S

**BILL OF RIGHTS**

These rights are the rights of every person who is asked to be in a medical research study. As a research participant, I have the following rights:

1. I have the right to be told what the research is trying to find out.

1. I have the right to be told about all research procedures, drugs, and/or devices and whether any of these are different from what would be used in standard practice.

1. I have the right to be told about any risks, discomforts or side effects that might reasonably occur as a result of the research.

1. I have the right to be told about the benefits, if any, I can reasonably expect from participating.

1. I have the right to be told about other choices I have and how they may be better or worse than being in the research. These choices may include other procedures, drugs or devices.

1. I have the right to be told what kind of treatment will be available if the research causes any complications.

1. I have the right to have a chance to ask any questions about the research or the procedure. I can ask these questions before the research begins or at any time during the research.

1. I have the right to refuse to be part of the research or to stop at any time. This decision will not affect my care or my relationship with my doctor or this institution in any other way.

1. I have the right to receive a copy of the signed and dated written consent form for the research.

1. I have the right to be free of any pressure as I decide whether I want to be in the research study.

---------------------------------------------------

If I have any questions or concerns I can ask the researcher or the research assistant. I can also contact the Office of the Human Research Protection Program (OHRPP), which

helps protect research study participants. I can reach the OHRPP by calling

310-825-5344 from 8:00 AM to 5:00 PM, Monday to Friday or participants@research.ucla.edu. If I call this office and do not speak English or Spanish, I should have someone available who can interpret for me. I may also write OHRPP, 10889 Wilshire Blvd., Suite 830, Los Angeles, CA 90095-1406.

05/2021

**UCLA IRB incident log**


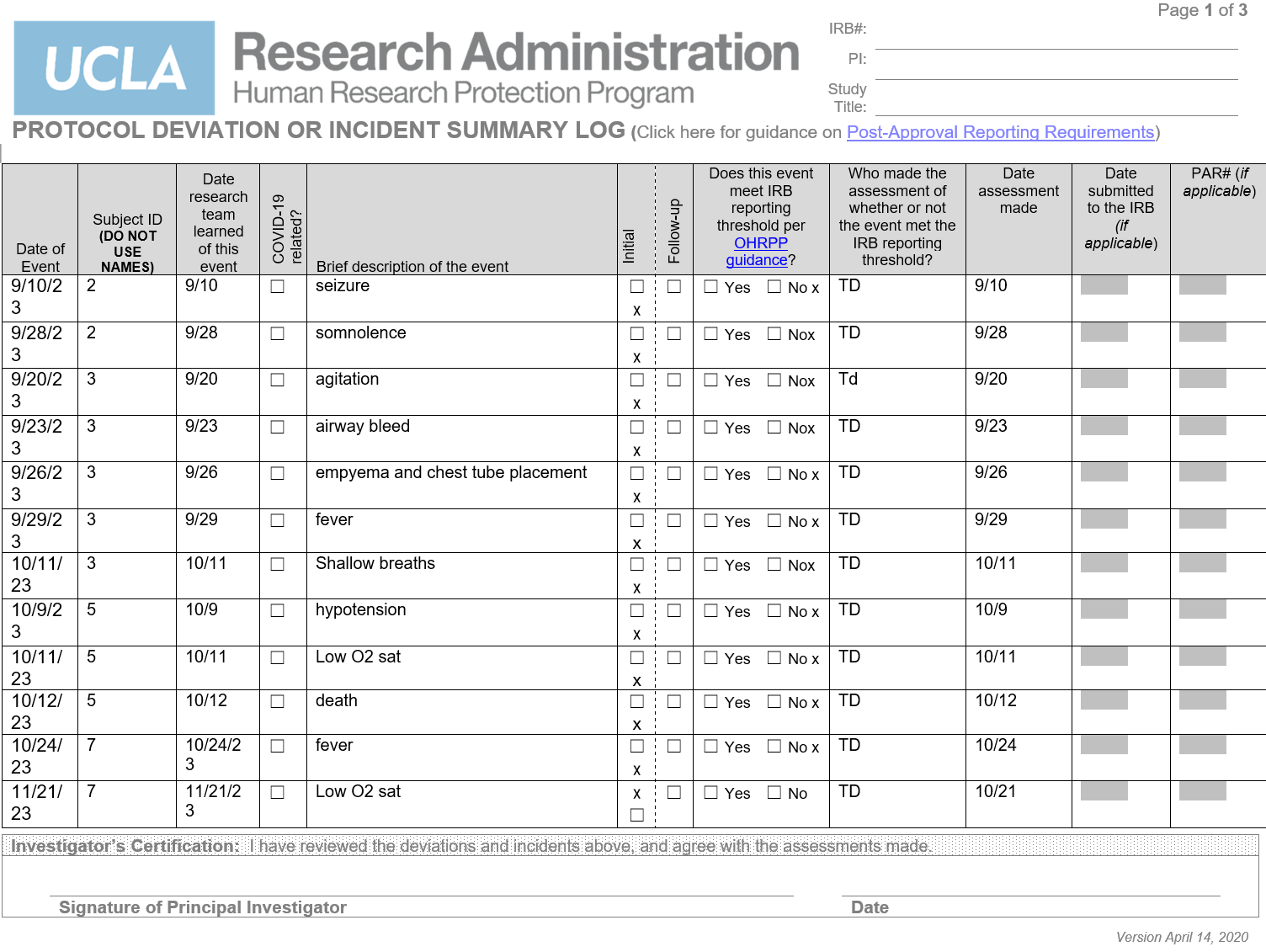


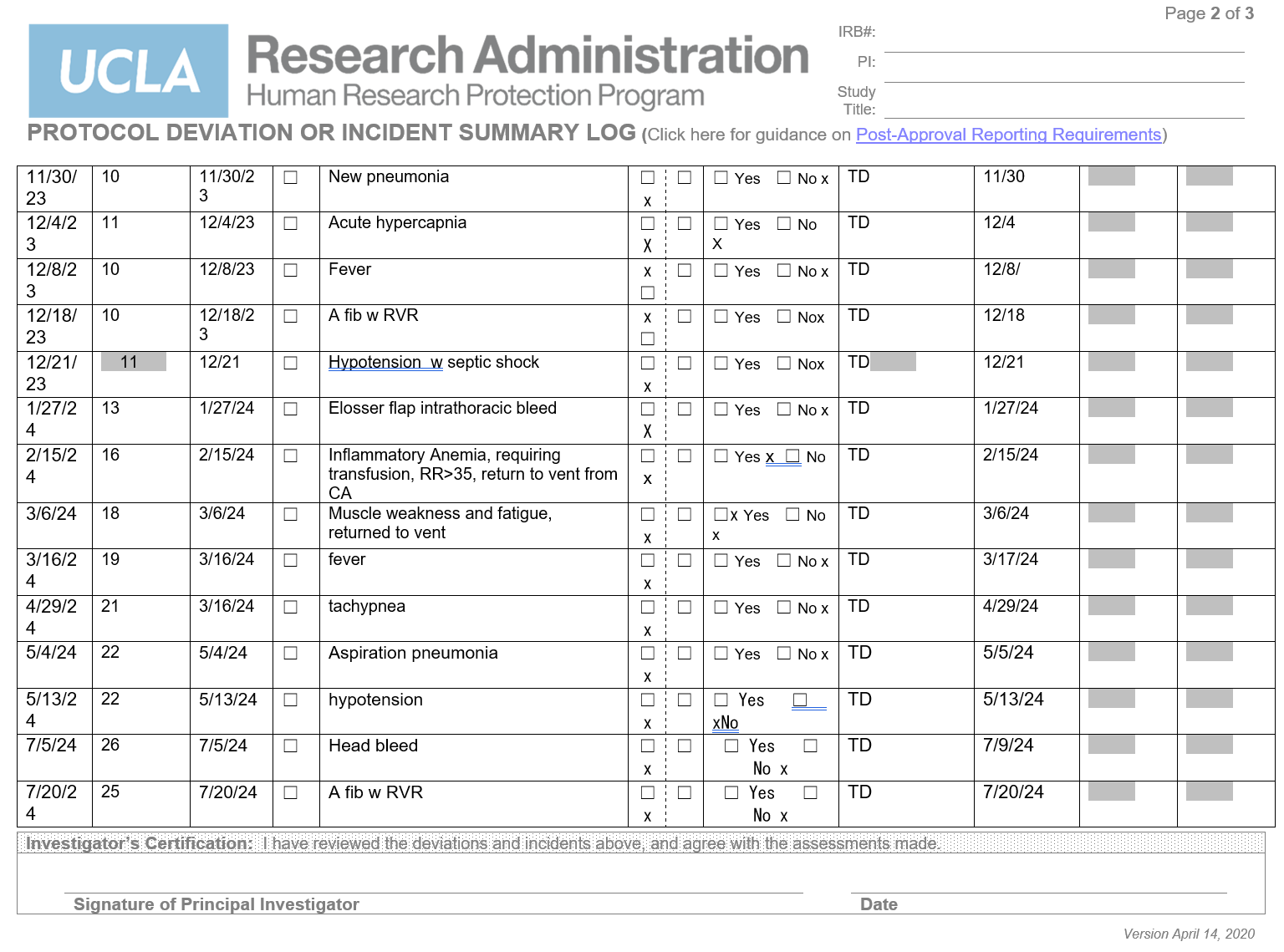


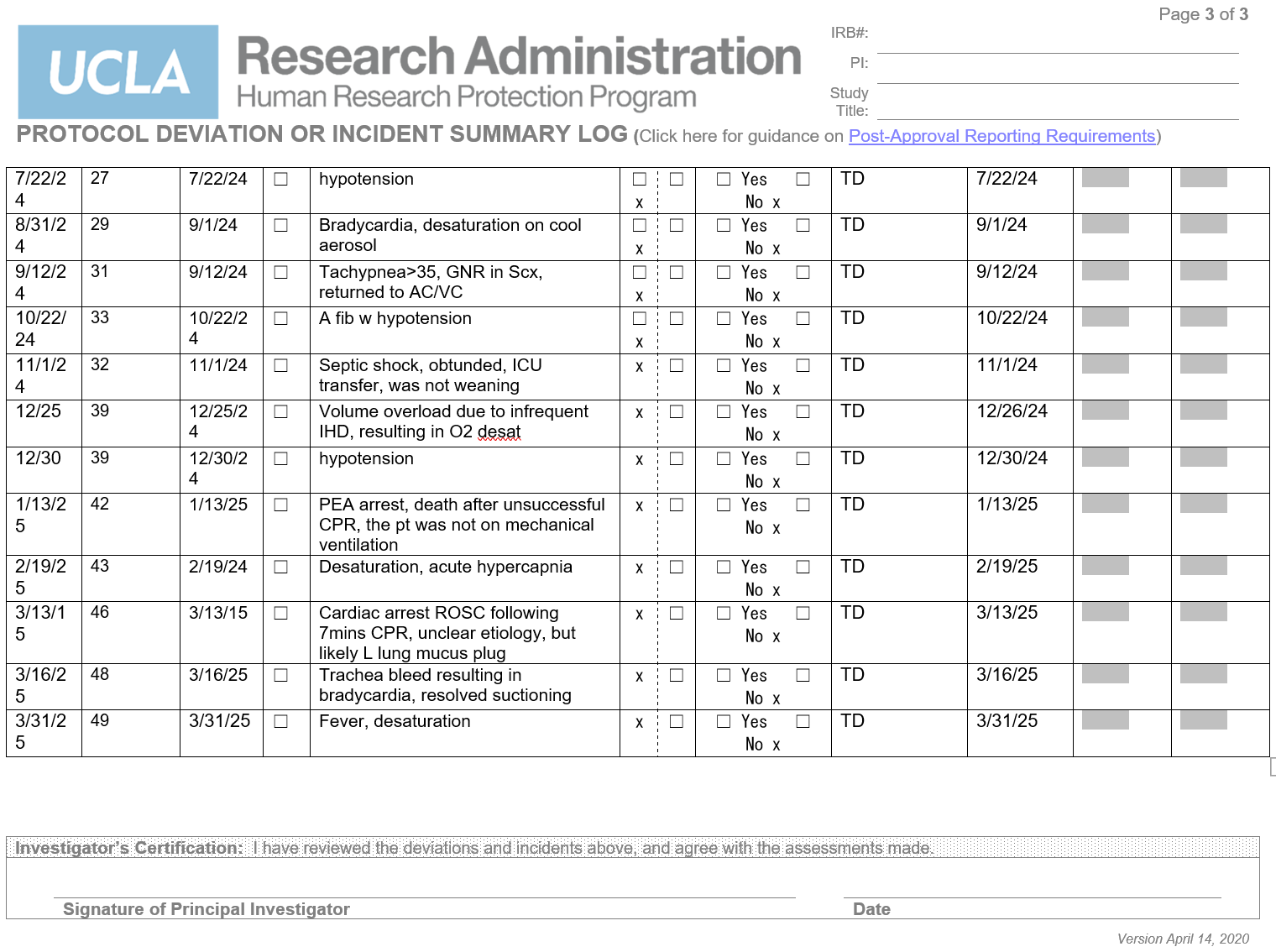


**UCLA IRB approval form**


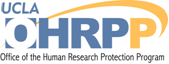
University of California Los Angeles

10889 Wilshire Blvd, Suite 830 Los Angeles, CA 90095-1406

<http://ora.research.ucla.edu/ohrpp>

General Campus IRB: (310) 825-7122 Medical IRB: (310) 825-5344

# APPROVAL NOTICE

**New Study**

**DATE:**

3

/

3/2023

**TO:**

TAMAS DOLINAY , MD

MEDICINE-PULMONARY DISEASE

**FROM:**

DANIEL CLEMENS, MD, PhD

Chair,

MIRB1

**RE:**

IRB#22-001420

Mechanical ventilator weaning in chronically ventilated patients

Version: Protocol v.1.0

The UCLA Institutional Review Board (UCLA IRB) has approved the above-referenced study. UCLA's Federalwide Assurance (FWA) with Department of Health and Human Services is FWA00004642.

## Submission and Review Information

Type of Review

Full Board Review

Approval Date

3

/

3/2023

Expiration Date of the Study

1/24/2024

Funding Source(s)

None

**Specific Conditions for Approval**

-- **UCLA Serving as IRB of Record** - The UCLA IRB has agreed to serve as IRB of

record for Barlow Respiratory Hospital. This may not be the only approval necessary to conduct this research. The relying investigator and/or relying institution remain responsible for determining that necessary approvals are in place and that the research may proceed.

-- **Translations Needed** - Please submit translated copies of your consent documents as an amendment(s) before recruiting or consenting any subjects for whom these translations are required.

-- **Research Participants Bill of Rights** - By California law, a copy of the Research Participants Bill of Rights in a language in which the participant is fluent must be given to all research participants in this study as there is a real or foreseeable risk of biomedical harm. Numerous translations are available for download on the HRPP website at <http://ora.research.ucla.edu/OHRPP/Pages/BillofRights.aspx>.

## Regulatory Determinations

-- **HIPAA General Waiver** - The UCLA IRB waived the requirement for HIPAA Research Authorization for screening.

**Documents Reviewed included, but were not limited to:**

Document Name

Document

Version #

[22](https://webirb.research.ucla.edu/WEBIRB/Doc/0/LQAF52DTM71KD7G2CAKNI83U8C/22-001420_Biomedical_ICF_WeaningPMV_clean_012723_TD.pdf)

[-](https://webirb.research.ucla.edu/WEBIRB/Doc/0/LQAF52DTM71KD7G2CAKNI83U8C/22-001420_Biomedical_ICF_WeaningPMV_clean_012723_TD.pdf)

[001420](https://webirb.research.ucla.edu/WEBIRB/Doc/0/LQAF52DTM71KD7G2CAKNI83U8C/22-001420_Biomedical_ICF_WeaningPMV_clean_012723_TD.pdf)

[_](https://webirb.research.ucla.edu/WEBIRB/Doc/0/LQAF52DTM71KD7G2CAKNI83U8C/22-001420_Biomedical_ICF_WeaningPMV_clean_012723_TD.pdf)

[Biomedical](https://webirb.research.ucla.edu/WEBIRB/Doc/0/LQAF52DTM71KD7G2CAKNI83U8C/22-001420_Biomedical_ICF_WeaningPMV_clean_012723_TD.pdf)

[_](https://webirb.research.ucla.edu/WEBIRB/Doc/0/LQAF52DTM71KD7G2CAKNI83U8C/22-001420_Biomedical_ICF_WeaningPMV_clean_012723_TD.pdf)

[ICF](https://webirb.research.ucla.edu/WEBIRB/Doc/0/LQAF52DTM71KD7G2CAKNI83U8C/22-001420_Biomedical_ICF_WeaningPMV_clean_012723_TD.pdf)

[_](https://webirb.research.ucla.edu/WEBIRB/Doc/0/LQAF52DTM71KD7G2CAKNI83U8C/22-001420_Biomedical_ICF_WeaningPMV_clean_012723_TD.pdf)

[Weanin](https://webirb.research.ucla.edu/WEBIRB/Doc/0/LQAF52DTM71KD7G2CAKNI83U8C/22-001420_Biomedical_ICF_WeaningPMV_clean_012723_TD.pdf)

[g](https://webirb.research.ucla.edu/WEBIRB/Doc/0/LQAF52DTM71KD7G2CAKNI83U8C/22-001420_Biomedical_ICF_WeaningPMV_clean_012723_TD.pdf)

[PMV](https://webirb.research.ucla.edu/WEBIRB/Doc/0/LQAF52DTM71KD7G2CAKNI83U8C/22-001420_Biomedical_ICF_WeaningPMV_clean_012723_TD.pdf)

[_](https://webirb.research.ucla.edu/WEBIRB/Doc/0/LQAF52DTM71KD7G2CAKNI83U8C/22-001420_Biomedical_ICF_WeaningPMV_clean_012723_TD.pdf)

[clean](https://webirb.research.ucla.edu/WEBIRB/Doc/0/LQAF52DTM71KD7G2CAKNI83U8C/22-001420_Biomedical_ICF_WeaningPMV_clean_012723_TD.pdf)

[_](https://webirb.research.ucla.edu/WEBIRB/Doc/0/LQAF52DTM71KD7G2CAKNI83U8C/22-001420_Biomedical_ICF_WeaningPMV_clean_012723_TD.pdf)

[012723](https://webirb.research.ucla.edu/WEBIRB/Doc/0/LQAF52DTM71KD7G2CAKNI83U8C/22-001420_Biomedical_ICF_WeaningPMV_clean_012723_TD.pdf)

[_](https://webirb.research.ucla.edu/WEBIRB/Doc/0/LQAF52DTM71KD7G2CAKNI83U8C/22-001420_Biomedical_ICF_WeaningPMV_clean_012723_TD.pdf)

[TD.](https://webirb.research.ucla.edu/WEBIRB/Doc/0/LQAF52DTM71KD7G2CAKNI83U8C/22-001420_Biomedical_ICF_WeaningPMV_clean_012723_TD.pdf)

[pdf.](https://webirb.research.ucla.edu/WEBIRB/Doc/0/LQAF52DTM71KD7G2CAKNI83U8C/22-001420_Biomedical_ICF_WeaningPMV_clean_012723_TD.pdf)

[pd](https://webirb.research.ucla.edu/WEBIRB/Doc/0/LQAF52DTM71KD7G2CAKNI83U8C/22-001420_Biomedical_ICF_WeaningPMV_clean_012723_TD.pdf)

[f](https://webirb.research.ucla.edu/WEBIRB/Doc/0/LQAF52DTM71KD7G2CAKNI83U8C/22-001420_Biomedical_ICF_WeaningPMV_clean_012723_TD.pdf)

0.01

***Important Note:*** Approval by the Institutional Review Board does not, in and of itself, constitute approval for the implementation of this research. Other UCLA clearances and approvals or other external agency or collaborating institutional approvals may be required before study activities are initiated. Research undertaken in conjunction with outside entities, such as drug or device companies, are typically contractual in nature and require an agreement between the University and the entity.

## General Conditions of Approval

As indicated in the PI Assurances as part of the IRB requirements for approval, the PI has ultimate responsibility for the conduct of the study, the ethical performance of the project, the protection of the rights and welfare of human subjects, and strict adherence to any stipulations imposed by the IRB.

The PI and study team will comply with all UCLA policies and procedures, as well as with all applicable Federal, State, and local laws regarding the protection of human subjects in research, including, but not limited to, the following:

Ensuring that the personnel performing the project are qualified, appropriately trained, and will adhere to the provisions of the approved protocol,

Implementing no changes in the approved protocol or consent process or documents without prior IRB approval

(except in an emergency, if necessary to safeguard the well-being of human subjects and then notifying the IRB as

soon as possible afterwards),

Obtaining the legally effective informed consent from human subjects of their legally responsible representative, and using only the currently approved consent process and stamped consent documents, as appropriate, with human subjects,

Reporting serious or unexpected adverse events as well as protocol violations or other incidents related to the protocol to the IRB according to the OHRPP reporting requirements.

Assuring that adequate resources to protect research participants (i.e., personnel, funding, time, equipment and space) are in place before implementing the research project, and that the research will stop if adequate resources become unavailable.

Arranging for a co-investigator to assume direct responsibility of the study if the PI will be unavailable to direct this research personally, for example, when on sabbatical leave or vacation or other absences. Either this person is named as co-investigator in this application, or advising IRB via webIRB in advance of such arrangements.

**CONSORT 2025 checklist**


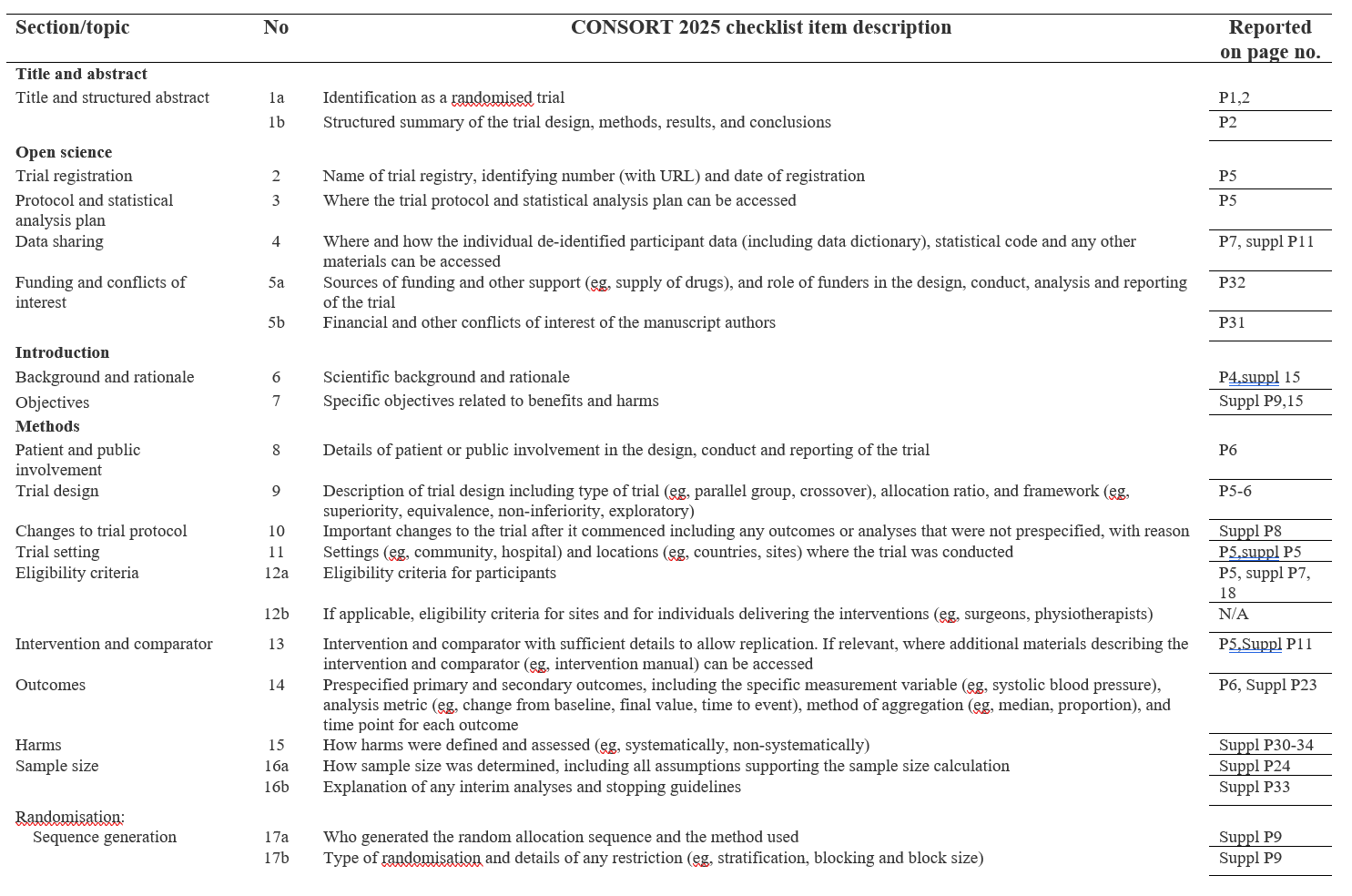


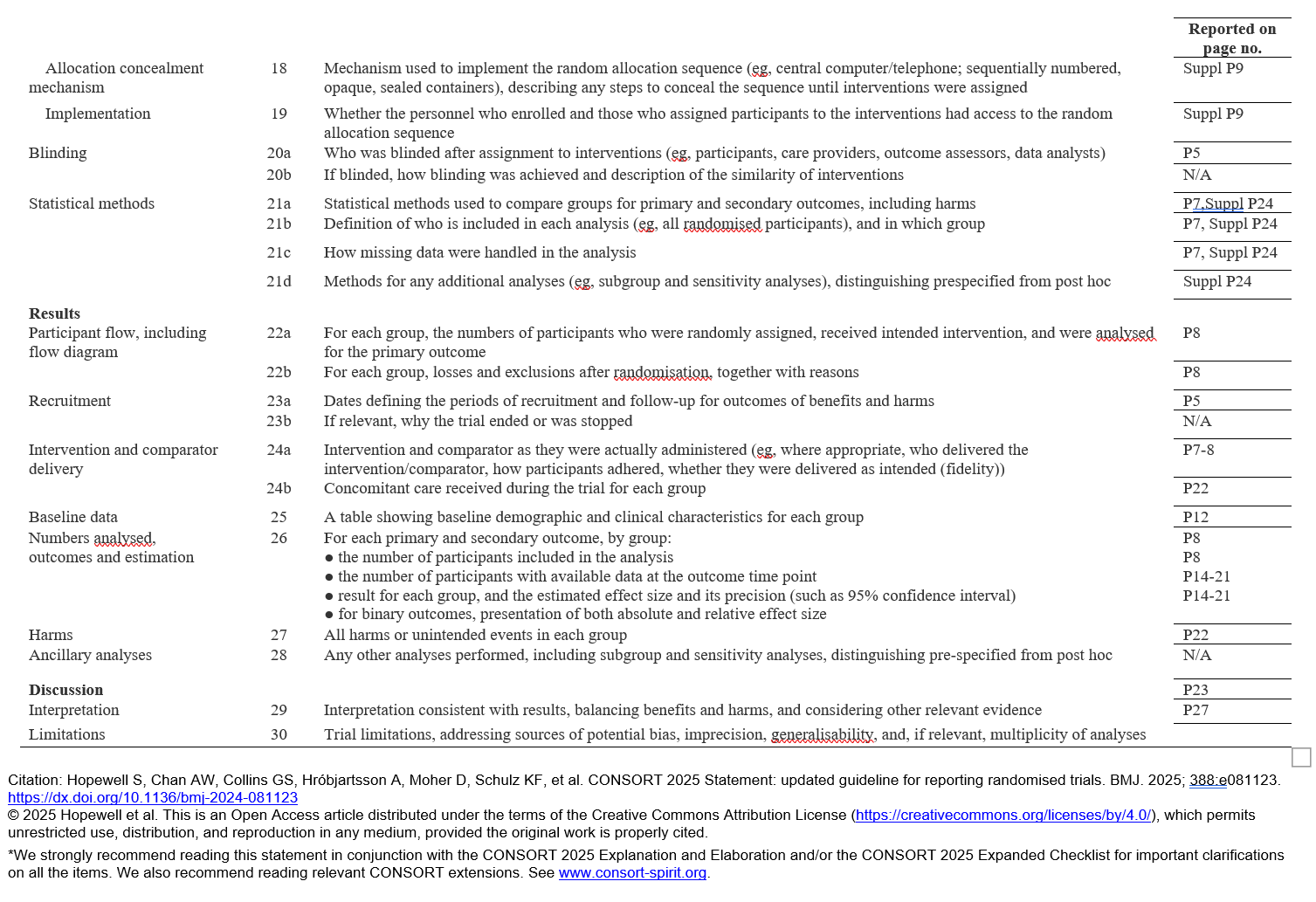

Supplement: Supplementary file 1 — Supplementary Material 1. [file 12890_2026_4341_MOESM1_ESM.docx]
